# The Intersection of Central Dopamine System and Stroke: Potential Avenues Aiming at Enhancement of Motor Recovery

**DOI:** 10.3389/fnsyn.2018.00018

**Published:** 2018-07-06

**Authors:** Annette Gower, Mario Tiberi

**Affiliations:** ^1^Ottawa Hospital Research Institute (Neuroscience Program), Ottawa, ON, Canada; ^2^University of Ottawa Brain and Mind Institute, Ottawa, ON, Canada; ^3^Departments of Medicine, Cellular and Molecular Medicine, and Psychiatry, University of Ottawa, Ottawa, ON, Canada

**Keywords:** dopamine receptors, amphetamine, L-DOPA, stroke recovery, pharmacotherapy, animal stroke models, clinical trials, motor recovery

## Abstract

Dopamine, a major neurotransmitter, plays a role in a wide range of brain sensorimotor functions. Parkinson's disease and schizophrenia are two major human neuropsychiatric disorders typically associated with dysfunctional dopamine activity levels, which can be alleviated through the druggability of the dopaminergic systems. Meanwhile, several studies suggest that optimal brain dopamine activity levels are also significantly impacted in other serious neurological conditions, notably stroke, but this has yet to be fully appreciated at both basic and clinical research levels. This is of utmost importance as there is a need for better treatments to improve recovery from stroke. Here, we discuss the state of knowledge regarding the modulation of dopaminergic systems following stroke, and the use of dopamine boosting therapies in animal stroke models to improve stroke recovery. Indeed, studies in animals and humans show stroke leads to changes in dopamine functioning. Moreover, evidence from animal stroke models suggests stimulation of dopamine receptors may be a promising therapeutic approach for enhancing motor recovery from stroke. With respect to the latter, we discuss the evidence for several possible receptor-linked mechanisms by which improved motor recovery may be mediated. One avenue of particular promise is the subtype-selective stimulation of dopamine receptors in conjunction with physical therapy. However, results from clinical trials so far have been more mixed due to a number of potential reasons including, targeting of the wrong patient populations and use of drugs which modulate a wide array of receptors. Notwithstanding these issues, it is hoped that future research endeavors will assist in the development of more refined dopaminergic therapeutic approaches to enhance stroke recovery.

## Introduction

Stroke refers to an interruption of blood supply to some regions of the brain. This can be either ischemic (about 85% of strokes) or hemorrhagic (about 15% of strokes) (Auriat and Colbourne, 2008). Stroke is a major cause of disability in adults. In 2008 stroke was the cause of 5.7 million deaths and 46.6 disability-adjusted life years worldwide (Oczkowski, 2013). Stroke survivors are left with damage to their brain resulting in various impaired domains of function. Among the most common of these is motor deficits, affecting up to 80% of patients (Langhorne et al., 2009). Currently available interventions for stroke include clot-busting approaches such as thrombolytic drugs or angioplasty. These interventions are only available in select cases and only in the hyperacute stage of stroke. Once the patient has stabilized, a combination of spontaneous recovery and rehabilitation allow them to regain some function. While rehabilitation methods such as physical therapy have been shown to be more useful than no rehabilitation at all, no one physiotherapy treatment has been shown to be more efficacious than the others (Oczkowski, 2013). Additionally, physical rehabilitation is only available to individuals who are well enough to complete it. Unfortunately, with currently available treatments, 40–60% of stroke patients still present with motor deficits in the chronic stage, after recovery has plateaued (Acler and Manganotti, 2013). There is a need for more strategies to enhance stroke recovery. One approach, pharmacotherapy, uses drugs and physical rehabilitation to boost recovery. Several classes of drugs have been investigated for this purpose, including selective serotonin reuptake inhibitors (SSRIs), cholinergic agents and dopamine (DA)-enhancing drugs (Rösser and Flöel, 2008; Viale et al., 2018). However, none of these therapies have been approved for routine clinical use in stroke recovery. Herein, we argue that interventions targeting the dopaminergic system hold particular promise. Indeed, stroke impacts the dopaminergic system, and this combined with the normal decline in DA function and motor learning abilities with age, makes it a relevant target. There is robust evidence in animal stroke models showing that DA-enhancing drugs can improve motor recovery, through a myriad of potential mechanisms that remain to be fully understood. Lastly, clinical investigations suggest that this may be a valid therapeutic tool for stroke survivors, although better selection of patient groups may be required. This review will focus on interactions between the dopaminergic system and the poststroke brain, specifically as it pertains to motor recovery. To this end, we have excluded evidence relating to therapeutic targeting of dopaminergic system in the facilitation of recovery from traumatic brain injury.

## Overview of the dopaminergic system

The dopaminergic system mostly consists of groups of midbrain DA-synthesizing neurons that enable a wide range of modulatory and neuroendocrine functions in the brain specifically mediated through four major pathways (Björklund and Dunnett, 2007; Brunelin et al., 2013; Grattan, 2015). The nigrostriatal pathway arises from DA neurons originating in the substantia nigra pars compacta (SNc) that primarily project their axons to the striatum to control motor activity. The mesolimbic and mesocortical pathways (a.k.a. the mesocorticolimbic pathway) stem from DA neurons located in the ventral tegmental area (VTA) that send axonal projections to the limbic (e.g., nucleus accumbens, amygdala, hippocampus) and cortical areas (e.g., prefrontal cortex, motor cortex, and somatosensory cortex), respectively. These two dopaminergic pathways play a critical role in reward, memory, learning, cognition as well as planning, control and execution of voluntary movements. The fourth pathway, typically referred to as tuberoinfundibular pathway, arises from DA neurons in the hypothalamic periventricular and arcuate nuclei that display anatomically distinct projections to the medium eminence of the hypothalamus, a region from which DA is released in the hypophyseal portal system to inhibit prolactin secretion from the anterior pituitary gland (Björklund and Dunnett, 2007; Brunelin et al., 2013; Grattan, 2015).

Once released DA interacts with six transmembrane G protein-coupled receptors (GPCRs) separated classically into the D_1_-class (D_1_R, D_5_R) and D2-class (short and long isoforms of D_2_R, D_3_R and D_4_R) subtypes, activating and inhibiting adenylyl cyclase, respectively. Aside from their canonical G_s/olf_ and G_i/o_-linked primary signaling pathways, studies have suggested a number of alternate G protein-dependent and independent signaling routes and downstream effects for these receptors (Beaulieu and Gainetdinov, 2011). There are many drugs which can modulate the activation of these receptors in a nonspecific and class-specific fashion but none exist that can bind to only one subtype. Studies in both animal and human stroke have mostly been done using indirect dopamine agonists, mainly amphetamine (AMPH), methylphenidate (MPH), and the DA precursor levodopa (L-DOPA). AMPH increases the release, and prevents the reuptake of DA, norepinephrine (NE) and to a lesser extent serotonin (Barbay and Nudo, 2009). MPH works by blocking the reuptake and thus increasing extrasynaptic DA and NE levels, although not as dramatically as AMPH (Kuczenski and Segal, 1997; Challman and Lipsky, 2000). Unlike DA, L-DOPA is able to cross the blood brain barrier. It can be given systemically and, once it enters the brain it is converted to DA (Barbeau et al., 1972). The DA molecule is in the synthesis pathway for NE, so L-DOPA can increase NE levels, however it has been reported that only 5% of L-DOPA ends up as NE, although this finding remains to be further validated (Nutt and Fellman, 1984). Notwithstanding this, it is worth mentioning that there is evidence supporting the direct contribution of noradrenergic neurons to dopaminergic neurotransmission through either NE acting as a DA receptor agonist or through co-release with DA (Vanderheyden et al., 1986; Devoto and Flore, 2006; Kubrusly et al., 2007; Smith and Greene, 2012; Root et al., 2015; Kempadoo et al., 2016; Takeuchi et al., 2016). Hence, indirect dopaminergic agonists such as AMPH related drugs may likely also mediate activation of DA receptors through their effects on noradrenergic axonal terminals.

## Effect of stroke on functioning of the dopaminergic system

Stroke impacts the DA system in a variety of ways, the implications of which are still somewhat unclear. For obvious reasons this is a difficult topic to study in humans, especially in the acute phase. In animal studies, differences in species and model can shift the timeline and magnitude of results, and this can make it difficult to translate these findings to humans. Add to this the importance of considering not just levels of DA and its metabolites [3,4-dihydroxyphenylacetic acid (DOPAC) and homovanilic acid (HVA)], but also levels of enzymes regulating DA synthesis (tyrosine hydroxylase or TH, and DOPA-decarboxylase) and degradation [monoamine oxidases MAO-A and MAO-B, and catechol-O-methyltransferase (COMT)], DA reuptake (dopamine transporter or DAT) and receptor proteins. Further complicating matters is that in the wake of stroke, any of these factors could be up or downregulated as part of the damage or as part of intrinsic attempts at recovery, making it difficult to determine how best to respond to these natural changes. Nonetheless, the background of the DA system remains an important consideration for those looking to modulate it to promote motor recovery from stroke.

### Stroke triggers early, massive dopamine release into the striatum

A massive DA release into the ipsilateral striatum almost immediately following the onset of ischemic stroke in the middle cerebral artery (MCA) territory has been repeatedly observed in many experimental models (Kogure et al., 1975; Ahagon et al., 1980; Brannan et al., 1987; Globus et al., 1988; Kawano et al., 1988; Slivka et al., 1988; Akiyama et al., 1991; Delbarre et al., 1992; Richards et al., 1993; Hashimoto et al., 1994; Toner and Stamford, 1996). Although this finding has been remarkable consistent, dissenting evidence does exist (Cvejić et al., 1980). The return to DA baseline levels is likely dependent upon the model used and the time that the ischemic period lasts, seeming to range from 30 min after reperfusion in bilateral MCA occlusion (MCAO) models (Akiyama et al., 1991), to several hours after microsphere injection (Kogure et al., 1975), and to take longer in permanent ligation or occlusion methods than in transient methods. All the studies discussed here used stroke models which significantly impact the striatum, so it remains unclear if this is a general reaction to ischemia or only to ischemia affecting the striatum.

Evidence shows that this DA release is not caused by action potentials (Akiyama et al., 1991). It cannot be attenuated with antagonists for D_1_R, D_2_R or both (Hashimoto et al., 1994). Prior lesioning of the substantia nigra (SN) on the side of the stroke prevents this massive DA release (Globus et al., 1987, 1988; Hashimoto et al., 1994). There is evidence which links the DA response to ischemia to local release of nitric oxide (Weinberger, 2002). Levels of DA metabolites DOPAC and HVA decrease during ischemia but rise after, which likely indicate efforts to return to baseline DA levels (Globus et al., 1988; Kawano et al., 1988; Slivka et al., 1988). DAT is overwhelmed during this release and drugs targeting it are unable to modulate the level of DA efflux, however it does seem to be active in the return to baseline (Akiyama et al., 1991; Toner and Stamford, 1996). Evidence suggests that the magnitude of the DA efflux correlates with the severity of the ischemia (Richards et al., 1993).

There appears to be differences in the degree of this response between animals of different ages, with aged animals having a more dramatic DA release after infarct and a more severe outcome from stroke (Delbarre et al., 1992). It has been suggested that the massive DA release contributes to neurodegeneration over the course of ischemia, as prior lesions to the SNc were observed to be neuroprotective in the striatum in several studies (Globus et al., 1987, 1988; Buisson et al., 1992; Weinberger, 2002). Additionally, use of D_2_-class antagonists, though not D_1_-class antagonists, have been reported to have a protective effect on neurons in the striatum (Hashimoto et al., 1994; Okada et al., 2005; Yulug et al., 2006a,b). In light of the well-established role of excessive glutamate release and sustained activation of extrasynaptic glutamate N-methyl-D-aspartate receptors (NMDAR) in mediating excitotoxicity and cell death during ischemic stroke, it remains unclear how the reported complex functional interactions between NMDARs and DA receptors contribute to either mitigating or exacerbating their deleterious effects (Wang et al., 2012; Brassai et al., 2015; Amantea and Bagetta, 2017).

The massive DA release is followed by a period of reduced DA levels in the hemisphere ipsilateral to the stroke, appearing as early as 2 h after stroke, or as late as 24 h (Zervas et al., 1974; Harrison et al., 1979; Ahagon et al., 1980). Some studies have suggested a loss or inefficiency in DA nerve terminals, with animals with more severe strokes having 30%, and moderately affected animals having 66%, of the number of DA nerve terminals of the contralateral hemisphere (Weinberger et al., 1983). In a rat photothrombosis model employing less intensive but longer duration irradiation to simulate a penumbra zone, levels of MAO-B were increased ~3-fold in the penumbra as compared to contralateral tissue at 4 h poststroke and ~2-fold at 24 h poststroke (Uzdensky et al., 2017). DOPA decarboxylase and TH were down regulated. Additionally, DAT levels were lower than the contralateral side (Uzdensky et al., 2017). These regulatory changes in the penumbra suggest lower DA levels are available after the immediate stroke period. An attenuated DA release response to high K^+^ stimulation, which was at about 40% of the response of non-ischemic controls, and was not modulated by nomifensine (a DAT inhibitor) treatment, has also been observed. This attenuation lasted at least 48 h after stroke, but was corrected by 98 h poststroke (Akiyama et al., 1991). In the contralateral hemisphere the concentration of DA, but not DA metabolites, has been found to be higher than sham operated animals at 1 week poststroke, but not at 2 weeks after stroke in a mouse photothrombosis model (Obi et al., 2018). It is clear that the massive DA release during the ischemia period leaves the DA system altered, which must be taken into account when considering the use of DA-enhancing therapies following stroke.

There is no data in humans regarding DA levels immediately following stroke, however two studies have used non-human primates. One study injected a shower of microemboli followed by one large embolus into the MCA of baboons. Animals were sacrificed 1 h after injection. DA was found to be increased in the cortical gray matter, significantly so in the frontal and occipital regions, which would be largely spared in this model. DA was found to be decreased in the caudate nucleus (Ishihara et al., 1979). Additionally, in squirrel monkeys subjected to MCA ligation and sacrificed after 3 h, there was a decrease in hemispheric DA on the side ipsilateral to the stroke as compared to the contralateral side (Zervas et al., 1974). It is possible that these decreases came after an initial increase in DA much sooner after stroke, like what is seen in rodent models. In the absence of earlier data, it is difficult to draw a conclusion as to whether primate stroke follows the same pattern as rodents, though it does seem certain that the DA system is modulated in the wake of stroke.

### Evidence indicates a decrease in DA receptors following stroke

Less information is available regarding the effect of stroke on levels of DA receptors.

Several studies suggest reduced D_2_R expression in the hemisphere ipsilateral to the stroke around 2–14 days after stroke (Dawson et al., 1994; Martín et al., 2013; Sieber et al., 2014). Less clear are the levels of D_1_R expression. In two studies employing the MCAO model, D_1_R expression decreased in the ipsilateral hemisphere, with evidence indicating this was due to down regulation and not simply loss of D_1_R expressing cells (Abe et al., 2004; Sieber et al., 2014). Meanwhile, a study using a rat photothrombotic model found a decrease in D_1_R levels in the infarct core but not in the penumbra (Rogozinska and Skangiel-Kramska, 2010). A transcriptional analysis in a transient MCAO model in mice found two subsets of differentially expressed genes responding to stroke in age-independent and dependent manner (Sieber et al., 2014). Furthermore, genes that were regulated differently following stroke in an age-dependent manner showed greater down- (or up-) regulation of gene response to stroke, including genes related to dopaminergic function (*Drd1* and *Drd2*), in younger animals (Sieber et al., 2014).

### Striatal ischemia leads to development of secondary exofocal degeneration in the substantia nigra pars compacta (SNc) and pars reticulata (SNr)

Another well documented phenomenon is the delayed degeneration of the ipsilateral SNc following striatal stroke. Studies suggest a progression of events from the time of stroke to the delayed degeneration. Unfortunately, not all animal studies with the transient or permanent MCAO model have reported the percent loss of neurons in SNc and SNr after striatal ischemia. Nonetheless, the few studies reporting this metric have shown a nigral cell loss ranging from 25 to 50% (Zhao et al., 2001; Rodriguez-Grande et al., 2013; Prinz et al., 2015). Intriguingly, the delayed ipsilateral degeneration in rodent models following striatal ischemia systematically happens and is more extensive in SNr relative to SNc; an observation that seems different from antedate postmortem human studies clearly showing delayed nigral cell loss in SNc following a massive stroke in basal ganglia (Forno, 1983; Ohara et al., 1989; Tamura et al., 1990; Yamada et al., 1996). However, these postmortem studies are unclear about whether a cell loss is also a hallmark of human SNr (Forno, 1983; Ohara et al., 1989). Interestingly, a loss of GABAergic inputs to the SNr has been proposed to underlie the exofocal degeneration (Yamada et al., 1996; Zhao et al., 2001). The loss of inhibitory inputs results in “burn out” of the cells in the SN leading to neuronal swelling and the start of apoptosis processes, which are visible in some cells as early as 24 h poststroke (Zhao et al., 2002; Rodriguez-Grande et al., 2013). This development leads to the gathering of microglia and astrocytes in the region (Rodriguez-Grande et al., 2013; Prinz et al., 2015). The cellular edema, due to the increase in inflammatory immune processes and cellular swelling is visible on T2 weighted magnetic resonance imaging (MRI) as early as 4 days poststroke in animal models (Zhao et al., 2001, 2002; Kronenberg et al., 2012; Prinz et al., 2015).

Sometime after the developing degeneration is visible using MRI, a decrease in TH positive cells is observed in the SNc. Some studies have found this decrease to be transient and independent of cell loss (Yamada et al., 1996; Soriano et al., 1997), whereas others have seen it last for the duration of the experiment, and have linked it to loss of SNc neurons (Zhao et al., 2001, 2002; Winter et al., 2005; Kronenberg et al., 2012; Rodriguez-Grande et al., 2013; Prinz et al., 2015). Two studies have linked the exofocal degeneration to a decrease in striatal DA and to changes in behavior (Winter et al., 2005; Kronenberg et al., 2012). Winter et al. (2005) found right side ischemia led to a decrease in DA and HVA in both the left and the right striatum, as well as increased activity in behavioral testing. Left-side infarcted animals showed a reduction in striatal DA on the left side only, and showed increased anxiety-like behaviors. In this study the degeneration occurred at 10 weeks poststroke, much later than what most other animal studies have shown (Winter et al., 2005). Furthermore, Kronenberg et al. (2012) found that left, but not right MCAO led to depression-like behavior at 12 weeks poststroke, unless they were treated with SSRIs starting from 7 days poststroke, when the exofocal degeneration was apparent on MRIs. This study also demonstrated degeneration of VTA (Kronenberg et al., 2012). The fact that both studies indicate differences in the deficit resulting from delayed SNc degeneration depending on the lateralization of the infarct is of note, and fits with the suggestion that left side strokes lead to a higher incidence of poststroke depression in humans (Stanfill et al., 2016). The neuroprotective effect of SSRIs in the study Kronenberg et al. (2012) gives hope to the possibility of preventing this secondary event when it is detected on MRI, although how SSRIs might interact with the proposed loss GABAergic inhibition theory is unclear.

Delayed SNc lesions following striatal infarcts have also been observed in human case studies (Kinoshita et al., 2002), examinations of clinical populations (Nakane et al., 1992; Ogawa et al., 1997) and in postmortem human tissue analysis (Forno, 1983; Ohara et al., 1989; Ogawa et al., 1997; Zhang et al., 2012). In two separate studies of clinical populations looking at 18 and 25 stroke patients, all patients who had striatal damage had the appearance of T2 weighted hyperintensity in the ipsilateral SNc around 1–2 weeks after stroke. Theses hyperintensities were not present earlier after stroke, and became less intense over the ensuing months. No such hyperintensities were seen in patients with purely cortical stroke (Nakane et al., 1992; Ogawa et al., 1997). One of the patients with SNc hyperintensity died of pneumonia during the study period. Postmortem analysis of this individual revealed degeneration of the SNc ipsilateral to the side of stroke with marked neuronal loss, gliosis and a few macrophages, with no reactive neovascularization (Ogawa et al., 1997).

It remains unclear in the aforementioned studies if basal ganglia infarcts and stroke-induced SNc degeneration led to Parkinson's disease (PD)-like symptoms. Movement disorders are an infrequent problem following ischemic and hemorrhagic stroke, and if so progress into either hyperkinetic or hypokinetic conditions (Handley et al., 2009). Typically, when these conditions arise, they stem from neuronal damage in different locations of motor circuitry, but most usually from the basal ganglia and thalamus (Handley et al., 2009). Interestingly, one large observational study has reported an increased risk for a first-time diagnosis of PD following a previous stroke, and also the risk of a first-time ischemic stroke in recently diagnosed PD patients (Becker et al., 2010). Furthermore, striatal silent lacunar infarction may contribute to SNc degeneration and promote progression of PD (Rodriguez-Grande et al., 2013; Zhang et al., 2016). Meanwhile, it remains unclear if the increased risk of PD following stroke is linked, at least in part, to the vascular changes and ischemic neuronal injury brought by the cerebrovascular insult (Becker et al., 2010). Alternatively, coincidence of PD/parkinsonism and cerebrovascular disease in some patients may likely be explained by the higher prevalence of these conditions in aging population (Korczyn, 2015). Likewise, olfactory dysfunction is now recognized as a prodromal sign of many neurodegenerative diseases notably in PD patients, whose olfactory bulbs display a loss of mitral and tufted cells that relay odorant information to brain cortical areas (Doty, 2012; Cave et al., 2016). There are few literature cases of olfactory dysfunction in humans following stroke but none have reported cell loss in olfactory bulb (Rousseaux et al., 1996; Moo and Wityk, 1999; Wehling et al., 2015). Data obtained using transient forebrain ischemia in gerbils point to a differential cell vulnerability to ischemic insult, notably a delayed neuronal loss in glomerular and external plexiform cell layers while mitral cells are spared during the timeline studied (Koh et al., 2004; Her et al., 2007).

### Stroke can impact the response of the dopaminergic system to DA-modulating drugs

In a MCAO stroke model in rats 8 weeks or 5 months old at the time of stroke, ischemic animals showed significantly less of a catalepsy response to haloperidol (a non-selective D_2_R antagonist) as compared to age-matched sham animals. Young ischemic rats had the least catalepsy response at 2 h after drug injection, however they returned to the level of sham animals sooner than aged rats. In a test of locomotor activity following AMPH administration at 5 weeks poststroke, ischemic animals in both age groups reacted with a significantly greater increase in locomotor activity in response to AMPH, despite all groups having similar pre-drug locomotor activity levels. Again, the young ischemic animals had a more dramatic reaction than the older ones, but also returned to baseline levels more quickly, whereas the older ischemic animals were significantly more active than controls at the conclusion of the experiment, 2 h after AMPH administration. The aged and young control animals reacted similarly to AMPH. The authors hypothesize that lower levels of DA receptors due to cell loss in the striatum likely drove the attenuated response to haloperidol based antagonism, whereas a hyperresponsive mesolimbic system due to striatal damage, may have caused the enhanced reaction to AMPH (Borlongan et al., 1995). Interestingly, this pharmacological study shows a differential response of the DA system to stroke based on age, much like the transcriptional study discussed above (Sieber et al., 2014).

A recent study has also shown an altered response to drug manipulation of the dopaminergic system after stroke (Huang et al., 2017). In rats trained to consume alcohol prior to endothelin-1-induced (ET-1) stroke in the MCA territory, there was a greater preference for alcohol than that of sham animals. Huang et al. (2017) reported that spontaneous and stimulated firing of dorsomedial striatum (DMS)-projecting dopaminergic SNc neurons were higher in the side ipsilateral to stroke. They hypothesized that the aberrant behavior was due to a loss of dorsolateral striatal GABAergic projections to the SNc, releasing the inhibition on the DMS-projecting-dopaminergic SNc neurons (Huang et al., 2017). While this study may have many interesting implications for risks of alcoholism after stroke, it is unclear how this may relate to non-alcoholic patients, as the animals were pretrained to drink alcohol prior to stroke and the schedule of exposure to alcohol continued between non-testing days. Thus, these results may reflect the effect of chronic alcohol exposure after ischemia and not the natural condition of the DA system after stroke.

Most of the studies to date have used variations on MCAO in rodent models. This fact could be creating a misleading homogeneity in the results. Although strokes in the territory of the MCA are the most common in humans, many of these vary from the typical MCAO model stroke, and other stroke types do occur in humans. One human study with 48 stroke patients found that lesions in the brainstem correlated to decreases in DA levels and increases in HVA levels (indicating increased DA turnover), whereas cortical and striatal lesions were linked to increases in DA levels and decreases in DA turnover (Hama et al., 2017). These results must be interpreted with caution, as patients were tested at varying time points in the 3 months following stroke, while, as animal studies have shown, a given patient could have vastly different DA system landscape depending on the time point analyzed. DA and HVA levels were determined based on a 24 h urine sample, which does not necessarily perfectly represent brain levels (Hama et al., 2017). Nonetheless it reinforces the idea that different strokes may affect the DA system differently. Therefore, future animal work on the subject using more mobile and adaptable stroke methods such as endothelin-1 (ET-1) or photothrombosis will be required to test this. Lastly, more data regarding the state of the DA system following different strokes would help in deciding when and where therapies modulating DA systems are best applied.

## Animal studies on the effect of DA-enhancing drugs in poststroke motor recovery

A number of tests have been developed to evaluate motor and sensorimotor function in rodents. In many of these tests there is a measurable deficit in response to stroke induction with one or more of the various stroke models. A complete review of these tests is beyond the scope of this paper, and readers are directed toward other reviews (Durukan and Tatlisumak, 2010; Schönfeld et al., 2017). It is not entirely clear whether these behavioral tests are indicative of brain regions controlling specific motor tasks. In fact, it is generally agreed upon that these behavioral tests provide a broad metric of the sensorimotor function, for which the underlying brain connectivity patterns and neuronal circuits have yet to be fully established. Results of studies employing DA-enhancing drugs in poststroke motor recovery are summarized in Table 1.

**Table 1:** Summary of studies using AMPH or L-dopa in animal stroke models.

**Study**	**Model**	**Dosing/Rehab**	**Behavior**	**Outcomes**
Adkins and Jones, 2005	Cortical infarct caused by application of ET-1 to the surface of the rat cortex	1 mg/kg AMPH every third day starting 10–14 days postop, training given daily, 1–2 h after injection on injection days, on the single pellet reaching test	Single pellet reaching	AMPH rats were robustly better than saline mice while training continued, by 2 months after conclusion of training, AMPH mice had declined and the saline mice improved to the extent that the two experimental groups were similar
Alaverdashvili et al., 2007	Female rats, craniotomy followed by removal of pia mater and surface blood vessels	Oral D-amphetamine 1 mg/kg beginning 24 h poststroke given every 3rd day for 8 doses, half hour before training on their specific reaching task	Either single pellet reaching with trained trip to back of the box between reaches, modified single pellet or tray reaching	AMPH and controls were similar by the end of the experiment on all tasks, controls recovered faster than AMPH animals, having significantly better performance on some days, AMPH animals typically more qualitatively impaired than controls
Auriat and Colbourne, 2008	Collagenase intracerebral hemorrhage model, primarily striatal damage	2 mg/kg AMPH on days 7, 9, and 11, housed in an enriched environment and training on the tray reaching and beam walking tasks, 30 min after injection	Tested beam walking (non-aversive), a neural deficit score, the Montoya staircase, and the tray task, horizontal ladder	Effect of rehab but not the drug on beam walking and horizontal ladder, no effect on the Montoya staircase or the non-beam portion of the neural deficit score, tray task was not analyzed due to high number of animals which had to be excluded
Barbay et al., 2006	Adult squirrel monkeys, cauterization of surface blood vessels of microelectrode-determined hand representation and ~500 μm into arm representation	Single injection of 0.25 mg/kg AMPH 1 h before hand dexterity training on day 10 postop, training continued for 14 days	Kluver board (skilled reaching)	AMPH and rehab animals were significantly better than rehab alone animals on days 13,14, 17, 18, 19, 20, 21, and 22 poststroke, at 9 weeks postop AMPH was still significantly better than rehab controls
Boyeson and Feeney, 1991	Suction ablation of anterior and neocortical cerebellar cortex in rats	Starting 24 h after ablation 2 mg/kg AMPH, 0.4 mg/kg haloperidol, both or saline injections every 4 days for a total of 6 injections	Beam walking task	Recovery in cerebellum-lesioned rats not as complete as in motor cortex lesioned rats, Saline group recovered the most, haloperidol group recovered the worst, with a marked dip in performance after drug
Brown et al., 2004	Photothrombotic lesion in rats, selecting for highly impaired animals	2 mg/kg D-AMPH or saline given 1 day poststroke, with or without training on the beam and daily testing, or without daily testing	Beam walking without aversive stimuli	Training and daily testing both improved recovery, while AMPH slowed recovery, although AMPH and experience or training did recover fully by 10 days poststroke
Feeney and Hovda, 1983	Motor cortex ablation followed by packing the wound, in cats	5 or 8 mg/kg D,L-AMPH on days 4, 9, and 15	Tactile placing in response to paw stimulation	Placing response is weakly augmented about 3 h after administration on day 4 postop, at day 9 and day 15 saw a much larger restoration of tactile placing which lasts 12–24 h
Feeney and Hovda, 1983	Motor cortex ablation followed by packing the wound, in cats	5 mg/kg of D-AMPH, L-AMPH, D,L-AMPH or D,L-AMPH followed by haloperidol	Tactile placing in response to paw stimulation	In cats with ablations and no recovery racemic mixture was the most effective, D-AMPH had some effectiveness and L-AMPH had almost no effectiveness, haloperidol at 0.2 mg/kg was able to supress D,L-AMPH, and at 0.4 mg/kg was able to block it. In partially recovered cats but not unlesioned cats haloperidol was able to block tactile reaching
Gilmour et al., 2005	ET-1 stroke in rats	2 mg/kg AMPH, staring on day 2 postop and continuing every 3rd day until day 26 (8 days administration total)	Paw reach and foot fault, ipsilateral limb was bandaged to prevent use	No benefit of AMPH on the foot fault test, saw a benefit of AMPH on paw reaching 24 h after drug, recovery of AMPH animals still present 6 days after last injection
Goldstein, 2009	Ablation of fore and hindlimb sensorimotor cortex in rats	D-AMPH, haloperidol or saline for 5 days, rats are housed in an enriched environment and some are fitted with casts that prevent use of unimpaired forelimb 24 h before first drug dose	Cylinder test and beam walking (evaluating time to cross the beam)	On the cylinder test, among non-restricted animals AMPH animals performed best and haloperidol animals performed very poorly, restrictive casts offset the deficit in haloperidol animals and increased saline animal's performance to the level of AMPH+ restraint animals, no differences were seen between groups in the beam walking test
Goldstein and Davis, 1990b	Suction lesion to the level of the white matter	2.6 mg/kg D-AMPH	Beam walking with aversive stimulus, began 24 h after surgery and continuing until 48 h postop	Overall faster improvement of AMPH animals in one dose, however see AMPH non-responders and some saline animals spontaneously recovered
Goldstein and Davis, 1990c	Suction ablation of the cortex to the level of white matter in rats	2.6 mg/kg +D-AMPH, training is 6 trials on the beam walking task at 1 h intervals beginning 1 h after injection	Beam walking	At 24 h after injection AMPH+ training had significantly better performance on the beam walking task as compared to all groups, the training alone and AMPH alone groups were better than the saline group, but not significantly
Goldstein and Davis, 1990a	Rats, suction ablation of gray matter	2 mg/kg AMPH	Beam walking with aversive stimulus, given as massed or spaced trials	AMPH helped both massed and spaced trial rats, helped massed trials more early on
Hovda and Feeney, 1984	Cats, motor cortex ablation	5 mg/kg AMPH with experience, single dose or multiple doses days 10, 14, 18, and 22 after surgery	Beam walking and tactile placing	Fastest recovery with AMPH and experience, slower but complete recovery with AMPH alone (at 60 days post op) Saline group had incomplete recovery, no improvement on tactile placing test
Hovda et al., 1987	Cats, primary visual cortex ablations	5 mg/kg AMPH on days 10, 14, 18, and 22 postop	Tactile placing (eyes covered, stimulate hairs of dorsal surface)	AMPH cats show recovery within 3 h of first dose, recovery lasts to end of experiment (30 days)
Liu et al., 2011	Transient MCAO, lesions mainly in temporoparietal cortex	2 mg/kg AMPH every 3rd day for 4 weeks	Turning asymmetry during body swing test	AMPH animals had significantly less asymmetry on body swing, AMPH group had significantly better body posture while suspended on day 12
Papadopoulos et al., 2009	MCAO in rats, only cortical damage	2 mg/kg AMPH given on days 2, 5, and 8 postop with or without enriched environment (EE) or focussed activity (starting on day 2 and continuing twice a day for 3 weeks and then once a day for a further 5 weeks)	Forelimb reaching task performed daily, Monday-Friday for 8 weeks, and horizontal ladder performed weekly, behavior testing not done under the drug	AMPH combined with environmental enrichment and focussed activity was significantly better than all other groups on both skilled reaching and the horizontal ladder, show complete recovery on skilled reaching, AMPH and EE and AMPH with EE and focussed activity recovered completely
Ramic et al., 2006	Aspiration lesion of gray matter and some white matter damage	2 mg/kg D-AMPH on days 2 and 5 post-lesion, rehab under drug's effect, rehab involved a variety of forelimb involving climbing tasks and EE	Ladder walking, pellet reaching	AMPH + rehab group showed significant improvement at 1 week postop, AMPH only at 2 weeks postop on pellet reaching, see significant recovery at 1 week in combo group and 6 weeks in AMPH only on ladder walking, no significant benefit of rehab only
Rasmussen et al., 2006	Injection of autologous macro-clot to the middle cerebral artery in rats	3.5 mg/kg D-AMPH sulfate given on days 1, 3, 5, and 7 postop, physical therapy consisted of Montoya staircase for 15 min and T-maze for 20 runs on drug days	Montoya staircase	Animals given only therapy performed significantly better than controls and had no asymmetry, AMPH and AMPH + therapy animals still had asymmetry, AMPH + therapy was better than AMPH alone and AMPH alone was better than controls but not significantly so
Rasmussen et al., 2011	Embolic strokes in rats	No AMPH, early AMPH (3.5 mg/kg 10 min after embolization) Late AMPH with training (days 2, 5, 8, and 11 1 mg/kg with Montoya staircase) or both early and late AMPH	Montoya staircase, recovery assessed from days 14–25 poststroke	Acute AMPH group performed better than the control group on the Montoya staircase, the late AMPH and combination AMPH group both performed much worse than the controls
Ruscher et al., 2012	Transient MCAO in male rats	5 days of either 1, 5 or 20 mg/kg L-dopa (with benserazide) or placebo	Rotating pole test (various speeds), the cylinder test, a composite neuroscore	Significantly better improvement of the 20 mg/kg L-DOPA group as compared to controls on all speeds of the rotating pole test at 7 days post-infarct, the neuroscore at 7 and 14 days poststroke and the cylinder test 14 days poststroke. Significantly better improvement above the controls on the 5rpm speed of the rotating pole at 7 days poststroke and on the cylinder test at 14 days poststroke
Schmanke and Barth, 1997	Male rats with large unilateral electrolytic lesions of the sensorimotor cortex	24 h after surgery given either 2 mg/kg D-AMPH or saline	Beam walking, foot fault test, bilateral tactile stimulation (adhesive removal) test with neutralization, vibrissae > forelimb placing, forelimb > forelimb placing	Early beam walking recovery improved with AMPH, from 1–6 h post injection. On foot fault test, bilateral tactile stimulation test and vibrissae > forelimb placing and forelimb > forelimb placing there was no difference between the groups
Schmanke and Barth, 1997	Male rats with small unilateral electrolytic lesions in forelimb sensorimotor area	2 mg/kg D-AMPH at 1, 3, and 5 days postop	Beam walking, foot fault test, bilateral tactile stimulation (adhesive removal) test with neutralization, vibrissae > forelimb placing, forelimb > forelimb placing	AMPH mice showed recovery sooner on the beam walk and were significantly better on days 7, 9 and 15 postop. AMPH mice showed improvement sooner on the vibrissae > forelimb placing and forelimb > forelimb placing, no differences in foot fault test or bilateral tactile stimulation test
Schmanke and Barth, 1997	Male rats with electrolytic lesions of the caudal forelimb representation area.	2 mg/kg of D-AMPH 1, 3 and 5 days postop with or without forelimb placing training	Vibrissae > forelimb placing and Forelimb > forelimb placing	On vibrissae > forelimb placing AMPH + practice group performed the best starting about 10 days postop, AMPH alone and practice alone both performed better than saline, Forelimb > forelimb placing benefits from AMPH but not significantly, no improvement is observed until 35 days postop
Stroemer et al., 1998	Permanent MCAO in spontaneous hypertensive rats	2 mg/kg D-AMPH given on day 3, 6, and 13 poststroke and then every 3rd day until day 30, testing done 1 h after injection and again 24 h after injection	Foot fault test with large (6 cm) openings or small (3 cm) openings, Morris water maze (not discussed here), postural reflex test, somatosensory disengage behavior, rearing behavior and grip strength	No differences in postural reflex, somatosensory disengage behavior, rearing behavior, or grip strength. AMPH rats did significantly better than saline rats on the foot fault test from 2 days after surgery onwards on large foot fault test, saw a similar pattern on the small foot fault test
Sutton et al., 1989	Bilateral cortical ablation in cats, varying size of lesions	5 mg/kg AMPH on days 12, 16, and 20 after surgery	Beam walking with a 10-point scale, started 6 days after lesion	AMPH animals performed better than saline animals, although some AMPH animals were completely non-responsive to treatment and some saline animals display full spontaneous recovery
Wolf et al., 2014	Distal MCAO, affecting primarily the cortex, in rats	2 mg/kg D-AMPH on 2, 5, and 8 days, focused activity where animals are placed on climbing apparatuses for 20 min beginning 15 min after injection and EE	Skilled reaching test performed daily, horizontal ladder performed once a week	AMPH + rehab performed significantly better than all groups and no longer had a significant difference from the baseline performance on skilled reaching and the horizontal ladder at 8 weeks post infarct

*The table summarizes the study parameters and the outcomes of studies quoted in the main text, which involve administration of AMPH or L-DOPA. For a summary of studies involving other drugs, or intraventricular infusion approaches, please see Feeney et al. (1993)*.

### Behavioral paradigms show rapid improvement of motor skills

Studies have demonstrated effects of drugs which increase DA release, beginning during the period of drug administration. Many of those have explored the effects of AMPH on motor and sensorimotor recovery following brain injury since a beneficial effect of AMPH on righting-attempts in decerebrated cats was first observed in 1946 (Maling and Acheson, 1946).

#### Beam walk test

A large amount of work has been done using the beam walking test to evaluate AMPH facilitated recovery in rats and cats following brain damage/stroke. This finding has been robust, with accelerated recovery being seen in many studies, usually very quickly after AMPH administration (Feeney et al., 1982; Hovda and Feeney, 1984; Sutton et al., 1989; Goldstein and Davis, 1990a,b,c; Boyeson and Feeney, 1991; Schmanke et al., 1996). Feeney et al. (1982) showed that the benefit of AMPH could be blocked by restraining the animals during the active period of the drug, which suggested the importance of experience in conjunction with AMPH. Interestingly, the effect could be blocked by coadministration of haloperidol, indicating an important role for catecholamines, notably DA receptors (Feeney et al., 1982). It should be also noted that the effect of haloperidol alone in this study delayed the spontaneous biological recovery in rats with cortical damage but not in recovered control animals (Feeney et al., 1982).

Meanwhile, several studies did not show a benefit to AMPH treatment on performance on the beam walking task (Boyeson and Feeney, 1991; Brown et al., 2004; Auriat and Colbourne, 2008). Boyeson and Feeney (1991) performed cerebellar ablation lesions and found AMPH made the animals worse than saline controls, although none of the groups showed the same degree of spontaneous recovery seen in cortical ablation studies suggesting that gross motor deficits resulting from cerebellar lesions may have low rates of recovery. Similarly, the striatal lesions in the intracerebral hemorrhage model in the Auriat and Colbourne (2008) study were not responsive to this therapy. It may be that subcortical and cerebellar lesions are not responsive to this treatment; possibly these areas are the sites of action for the early AMPH effect seen in the other beam walking studies. Likewise, no benefit of AMPH on the beam walking task was also reported using a cortical photothrombosis model, which creates a very different injury than an ablation or electrolytic lesion model (Brown et al., 2004). The discrepant results obtained in these two rat studies may be explained in part by the different methodology for training on the beam walk, which did not use aversive stimuli (Brown et al., 2004; Auriat and Colbourne, 2008). Instead of aversive stimuli, as is typically used, the animals were trained by being placed successively farther from the goal box.

Further work in the rat beam walking paradigm shows this effect can be seen with the AMPH related drugs MPH, phentermine and phenylpropanolamine (Feeney et al., 1993). Some studies show enhancement of recovery with NE alone, administered via interventricular infusion or transplant of adrenal medulla cells (Boyeson and Feeney, 1990; Feeney et al., 1993). Similar interventricular infusion experiments, as well as work with the mixed D_1_R/D_2_R agonist apomorphine, did not indicate a prominent role for DA in this fast-acting effect on beam walking ability. It seems likely that the role of the DA system becomes more obvious given multiple administrations, and a longer time course. In the rat beam walking paradigm, α_1_-adrenergic receptor (α_1_AR) antagonists can inhibit the AMPH mediated enhancement of motor recovery, although α_1_AR agonists were not found to be sufficient to reproduce this recovery. The α_2_-adrenergic (α_2_AR) agonists slowed recovery, while antagonists could facilitate it. These findings seem to contradict the theory that the effect of AMPH is mediated solely through NE. Interestingly, desipramine, which blocks NE reuptake but is not a stimulant, is able to facilitate recovery, while MK-801, which blocks NE reuptake but is also an NMDA receptor antagonist, cannot (Feeney et al., 1993).

Although work in this paradigm has provided valuable insights and directed more recent work, it should be noted that results on this test are reported using an ordinal scale, which spans a wide range of capabilities in relatively few categories. On the two lowest levels of the scale the rat does not cross the beam at all. Performing the task with aversive stimuli increases the stress of the task for the animal. The aversive stimuli and AMPH may intersect to affect the way the animal experiences practice sessions, perhaps inducing animals to be more active and to respond more often to the stimuli and perform the test, getting more practice on the task. It should also be noted that prodding rats forward during the beam walking task was seen to improve performance in lesioned rats during both massed and spaced trials, indicating that motivational factors may be important on this task (Goldstein and Davis, 1990c). Furthermore, attempts to replicate positive effects of AMPH on beam walking in more modern stroke models which employ ischemia or hemorrhage, have not been successful.

#### Tactile placing in cats

Another classic paradigm for testing the effect of AMPH on recovery from stroke is testing tactile placing after stimulation of the dorsal paw surface in cats with sensorimotor cortex ablations. Recovery and reaction to AMPH seems to be determined by the lesion location. Large prefrontal cortex lesions never show recovery regardless of AMPH administration (Hovda and Feeney, 1984). Lesions affecting primarily the visual cortex, showed early and lasting complete restoration after injection with AMPH. Like in beam walking this effect could be blocked with haloperidol (Hovda et al., 1987). A study affecting the motor cortex led to permanent deficits in paw placing in most animals, but exposure to AMPH could temporarily restore tactile placing. Animals tested at 4 days post lesion had very minimal and short-lived recovery of the response at 3 h after the drug. Animals tested at 9 and 15 days after lesioning had much more robust responses that lasted about 12 h after administration of AMPH. In partly recovered cats, but not unlesioned cats, this recovery could be blocked with haloperidol (Feeney and Hovda, 1983). Due to the early onset of recovery, and the ability for the animal to respond positively to AMPH at 4 days postop, a time thought to be too soon for many plasticity and sprouting processes, these results have been taken to indicate that a rapid response to the drug is involved. However, the weakness of the response at 4 days postop may suggest that while AMPH may be of some benefit that early, it becomes more powerful at later time points when slower acting recovery mechanisms may have come into play. Again, haloperidol was able to block the benefit of AMPH. Interestingly, a weaker and more transient, but still statistically significant, increase in tactile placing was seen in cats given 0.25 and 0.5 mg/kg apomorphine. The use of higher and lower doses showed no effect (Feeney and Hovda, 1983).

#### Behavioral paradigms demonstrate recovery after a delay

In several motor behavioral tasks showing recovery mediated by AMPH, L-DOPA or VTA stimulation, recovery was shown not during the first period of administration, but after a delay. In these studies repeated administration was used.

#### Tactile placing in response to vibrissae or forelimb stimulation in rats

A paradigm similar to tactile placing in cats has been used in rats, this time with more lasting recovery. The paw placing response to vibrissae stimulation or forelimb stimulation can recover in rats with small electrolytic lesions of the motor cortex, given three administrations of AMPH. Rats recover sooner and more completely on placing in response to vibrissae stimulation, than they do on forelimb stimulus-forelimb placing (Schmanke et al., 1996; Schmanke and Barth, 1997). In both vibrissae > forelimb and forelimb > forelimb paradigms performance that is significantly greater than vehicle controls appears after a delay (10 and 35 days, respectively) (Schmanke et al., 1996; Schmanke and Barth, 1997).

#### The foot fault test and the horizontal ladder

The foot fault test and the horizontal ladder test both involve an evaluation of the percentage of missteps an animal makes while traversing an elevated grid or ladder surface. While not a fine motor task, this task requires more refined motor control than the beam walking task (Barbay and Nudo, 2009). Among studies showing a benefit of AMPH on this test, all gave AMPH at least twice, indicating that more chronic stimulation may be more effective in this paradigm (Stroemer et al., 1998; Ramic et al., 2006; Papadopoulos et al., 2009; Wolf et al., 2014).

#### Skilled reaching tests

Many studies have also been done using skilled pellet reaching tasks in rats. Several studies have had success demonstrating accelerated or superior recovery on pellet reaching tasks when multiple AMPH administrations are paired with either training or focussed activity, although AMPH alone or paired with environmental enrichment also showed recovery above the level of controls in some studies (Adkins and Jones, 2005; Gilmour et al., 2005; Ramic et al., 2006; Papadopoulos et al., 2009; Wolf et al., 2014). In non-human primates following cauterization of surface vasculature in the hand representation, one dose of 0.25 mg/kg AMPH and 14 days of training initiated 10 days postop was able to significantly improve performance on a skilled reaching task as compared to controls on days 4, 5, and 8–13 of training. AMPH-treated monkeys were still significantly better than rehab only controls at 9 weeks postop (Barbay et al., 2006). The efficacy of a single dose of AMPH in a non-human primate model, when multiple doses are typically required in rodent models may be due to the differences in lesion type.

Among the studies that did not demonstrate a benefit of AMPH on skilled reaching recovery there are several possible factors (Rasmussen et al., 2006, 2011; Alaverdashvili et al., 2007; Auriat and Colbourne, 2008). Alaverdashvili et al. (2007) used an oral administration route, and used all female mice, which may have affected the drug bioavailability (Barbay and Nudo, 2009). Additionally, studies looking at recovery of the skill reaching task in rats with striatal damage showed no benefit to AMPH (Rasmussen et al., 2006, 2011; Auriat and Colbourne, 2008). These studies suggest that benefits gained from AMPH administration potentially depend on the location of stroke lesion.

#### Other behavioral tests

A single study, using long-term administration of 2 mg/kg AMPH in rats with MCAO lesions, saw reduced infarct size in the AMPH group and improvements in turning asymmetry and body posture in the body swing test (Liu et al., 2011).

Only one study in animals has demonstrated improved motor recovery on behavioral tests using L-DOPA treatment after stroke. In a MCAO stroke model, rats exhibited significantly better recovery on the rotating pole test at various speeds, the cylinder test and a neuroscore, when animals were given a high dose (20 mg/kg) of L-DOPA. On some tests improved recovery could be seen at the 5 mg/kg dose (Ruscher et al., 2012). As 5% or less of L-DOPA is converted to NE, this effect is very likely mediated by DA (Nutt and Fellman, 1984). In lesion studies on rats, VTA stimulation led to recovery of pre-lesion performance levels on a lever pressing to stop aversive stimuli task within 4 days, vs. no recovery seen in controls (Castro-Alamancos et al., 1992; Castro-Alamancos and Borrell, 1995). Data obtained with L-DOPA and VTA stimulation, in which the mechanism of action is most likely linked to the dopaminergic system, suggest that DA plays also a role in AMPH-triggered effects reported in aforementioned studies.

### Potential benefit of pairing forced use of the impaired limb and DA-enhancing pharmacotherapy

Forced use of the impaired limb by immobilizing the non-affected limb as part of training or during exposure to environmental enrichment, in conjunction with AMPH, showed improvement on the cylinder test, but not on the speed of beam crossing (Goldstein, 2009). In skilled reaching tests, animals were prevented from using their unimpaired limb during training and testing either by the setup of the apparatus, with success (Barbay et al., 2006) or via application of a tape-bracelet to prevent reaching through narrow slots (Alaverdashvili et al., 2007) without seeing recovery and by bandaging the ipsilateral limb during testing (Gilmour et al., 2005). These approaches lead to skilled reaching improvement but not improved recovery on the foot fault test. Overall, these studies suggest that there may be some benefit in combining constraint-induced movement therapy (CIMT) with DA-enhancing approaches.

## Potential mechanisms of action of DA-enhancing drugs

### Reversal of catecholamine diaschisis

Due to the rapid time course of response to AMPH on the beam walking paradigm, and the tactile placing test in cats, the dominant theory regarding the mechanism by which AMPH evokes benefits on these tasks is a resolution of diaschisis in depressed regions remote to the injury site. In cats given bilateral lesions of varying sizes, this improved recovery is still observed on the beam walking task. This demonstrates that the rapid improvement on beam walking is not dependent on the corresponding contralateral region, nor on the adjacent regions (Sutton et al., 1989). The resolution of diaschisis theory is supported by the finding that cytochrome oxidase activity, which provides a readout of cellular energy production, is supressed in rats with cortical ablations in a number of regions important for motor performance, but this suppression can be reversed with a single injection of 2 mg/kg AMPH 24 h after ablation (Sutton et al., 2000). Additionally, it has been shown that AMPH can attenuate traumatic brain injury-triggered remote reductions in cerebral metabolic glucose utilization (Queen et al., 1997). It may be that this rapid effect is more strongly mediated by NE than by DA, which would fit well with the findings mentioned above showing NE is more effective in the beam walking paradigm, and the weaker effect of apomorphine on tactile placing in cats. It is possible that a DA-mediated augmentation of recovery requires a longer time course to work, or perhaps requires repeated stimulation of the dopaminergic system to be fully apparent.

### Enhanced synaptic plasticity, axonal sprouting, and cortical map reorganization

#### Synaptic plasticity

The results implicating NMDA receptors in the early recovery response to AMPH indicate a role for long-term potentiation (LTP) processes in this rapid AMPH mediated recovery. AMPH has been shown to increase LTP in the hippocampus and the prefrontal cortex in non-injured rodents (Delanoy et al., 1983; Xu et al., 2010). Interestingly, in the prefrontal cortex AMPH-driven augmentation of LTP was primarily mediated by D_1_R, except in the case of hyperdopaminergic mice, where AMPH reinstated LTP via a recruitment of β-adrenergic receptors. Both of these receptors are coupled to the cyclic AMP (cAMP)-protein kinase A pathway (Xu et al., 2010). DA receptors have been shown to be important modulators of LTP in many parts of the brain, including PFC (Gurden et al., 1999, 2000; Huang et al., 2004; Wirkner et al., 2004; Hotte et al., 2005, 2006; Ruan et al., 2014), hippocampus (Otmakhova and Lisman, 1996; Lemon and Manahan-Vaughan, 2006) and striatum (Calabresi et al., 1997, 2000, 2007; Centonze et al., 2001, 2003; Dudman et al., 2003; Wolf et al., 2003; Shen et al., 2008). Most relevantly, DA receptors, in particular D_1_-class subtypes, have been heavily implicated in motor learning processes in both striatum and primary motor cortex (Willuhn et al., 2003; Luft et al., 2004; Willuhn and Steiner, 2006, 2008; Molina-Luna et al., 2009; Hosp et al., 2011; Rioult-Pedotti et al., 2015). Evidence from human studies of patients with Korsakoff's syndrome (McEntee et al., 1987), and in healthy human patients using transcranial magnetic stimulation (TMS) suggest that DA receptors play an important role in these processes in humans (Floel et al., 2005, 2008; Breitenstein et al., 2006; Meintzschel and Ziemann, 2006; Nitsche et al., 2006, 2009; Kesar et al., 2017).

#### Axonal sprouting

There is evidence implicating longer term processes for delayed recovery. Several studies which showed improvement on the foot fault and horizontal ladder test as well as skilled reaching tasks, saw improvement was associated with indicators of axonal sprouting and neurite growth. Stroemer et al. (1998) reported significant increases in growth associated protein 43 (GAP-43), a synaptogenesis marker, at 3 and 7 days relative to saline-injected animals. From day 14 postop to 2 month postop, AMPH-treated animals had increased distribution of synaptophysin, which also indicates synaptogenesis, on the side contralateral to the infarct (Stroemer et al., 1998). Biotinylated dextran amine neuroanatomical tracing was used to demonstrate significant increases in fibers projecting across the midline from the uninjured cortex at the level of the basilar pontine nuclei, red nucleus and cervical spinal cord in AMPH-treated animals (Ramic et al., 2006; Papadopoulos et al., 2009; Wolf et al., 2014).

Many of the studies discussed herein have shown recovery mediated by enhanced sprouting in the contralateral hemisphere (Ramic et al., 2006; Papadopoulos et al., 2009; Wolf et al., 2014) while there is evidence for recovery mediated by the infarcted hemisphere (Liu et al., 2011). However, the role of the contralateral hemisphere in motor recovery is a controversial one. Although the assumption of lost motor function by the intact hemisphere has been shown to improve outcome, this recovery mechanism is correlated with worse outcomes than the resumption motor control by the affected side (Boyd et al., 2017). It has been suggested that perhaps those who are more seriously afflicted may be more likely to undergo contralateral side-based recovery because their affected hemisphere is unable to recover. In such cases sprouting from the contralateral hemisphere may be the most powerful recovery mechanism available (Hallett, 2001; Jang, 2013).

#### Cortical map reorganization

In motor cortex ablation studies, immunostaining for c-Fos, a marker of neuronal activity and plasticity, indicated that the forelimb representations associated with lever pressing ability had relocated to the hindlimb after forelimb lesions, but only in rats receiving VTA stimulation during lever pressing (Castro-Alamancos et al., 1992). Lesions to the hindlimb area in recovered animals reinstated the post-ablation deficit on the forelimb lever pressing task (Castro-Alamancos et al., 1992). In a follow-up study, the forelimb motor area of rats was mapped before lesion and only the forelimb area targeted was in the lesion operation (Castro-Alamancos and Borrell, 1995). Again, better motor recovery was seen in rats subjected to VTA stimulation. Post-injury mapping of this group showed the forelimb motor area had moved to an area caudal of the lesion. Little to no forelimb area reorganization was seen in unstimulated animals. Thus, results obtained with VTA stimulation suggest a role for DA in cortical remapping processes.

An important issue with the use of lesioned animals in the above-mentioned studies is that they are not the best experimental models to study recovery following ischemic stroke. Interestingly, a study on somatosensory recovery following photothrombotic stroke found that poststroke administration of haloperidol impaired the rate of spontaneous recovery of the somatosensory system as compared to controls (Obi et al., 2018). The spontaneous recovery was linked to increased DA levels and astrocytic activity in the contralateral hemisphere. While mice in this model regain sensation spontaneously, recovery processes also lead to maladaptive plasticity, in the form of a shift to single neurons receiving stimulation from more than one limb, visible as early as 1 week after stroke, and fully developed by 4 weeks poststroke. Administration of partial D_2_-class agonist aripiprazole starting 2 weeks after stroke halted this process and resulted in significantly less neurons receiving input from all four limbs (Obi et al., 2018). These findings suggest that D_2_R may play a role in the prevention of maladaptive cortical map reorganization.

### Modulation of growth factor expression

#### Fibroblast growth factor 2 (FGF-2)

In addition to being a neuroprotective agent in stroke, FGF-2 (a.k.a. basic fibroblast growth factor) can act as an angiogenic factor to promote sprouting and neurogenesis in stroke models (Lin and Finklestein, 1997; Wada et al., 2003; Issa et al., 2005; Slevin et al., 2006; Paciaroni and Bogousslavsky, 2011). FGF-2 expression in VTA is augmented following AMPH administration (Mueller et al., 2006), which may play a role in poststroke recovery. Indeed, a study using the transient MCAO stroke model showed that AMPH-enhanced poststroke motor performance of rats was accompanied with a transient increase in FGF-2 expression in layer V sensorimotor neurons in the unlesioned cortex at 2 weeks poststroke, along with enhanced axonal sprouting across the midline from the unlesioned cortex (Wolf et al., 2014). Additionally, the study reported that media from FGF-2 conditioned astrocytes were able to stimulate neurite outgrowth in primary cultured neurons, which was linked to activation of β- and α_1_-adrenergic receptors. However, the role of DA receptors was not tested (Wolf et al., 2014). This issue is of potential clinical relevance for dopaminergic pharmacotherapy in poststroke motor recovery as DA receptor activation increases FGF-2 levels (Reuss et al., 2000; Roceri et al., 2001; Fumagalli et al., 2003, 2006a; Li et al., 2006; He et al., 2008; Zhang et al., 2009).

#### Brain-derived neurotrophic factor (BDNF)

BDNF has been linked to enhanced motor recovery following stroke via increased sprouting, neuronal plasticity and neurogenesis, and has thus been implicated as a factor in the beneficial effects of rehabilitation and exercise (Kurozumi et al., 2004; Schäbitz et al., 2007; Ploughman et al., 2009; Mang et al., 2013; Berretta et al., 2014; Livingston-Thomas et al., 2016; Cook et al., 2017). AMPH administration during poststroke recovery in a transient MCAO rat stroke model leads to an increase in the levels of axonal fiber and white matter growth (ipsilateral side), neurofilament (ipsilateral and contralateral side), synaptophysin (ipsilateral side), matrix metalloproteinase 2 activity (ipsilateral side cortex), and mRNA for BDNF (ipsilateral side cortex) (Liu et al., 2011). These findings suggest an increase in sprouting processes in the infarcted cortex modulated by an AMPH-dependent BDNF expression. Interestingly, the infarct-induced reduction of BDNF mRNA levels in the lesioned cortex was attenuated by the poststroke AMPH treatment (Liu et al., 2011). Likewise, DA-enhancing drugs used in the treatment of Parkinson's disease (e.g., L-DOPA and MAO-B inhibitors) are also known to increase BDNF levels (Fumagalli et al., 2006b). Meanwhile, the mechanism underlying AMPH-induced BDNF increase during poststroke recovery is unclear, but likely involved the dopaminergic system. In fact, DA receptors can increase BDNF protein levels through D_1_R, D_5_R and D_1_R-D_2_R heterodimers or through D_1_R-mediated transactivation of tropomyosin receptor kinase B (TrkB), the BDNF receptor (Küppers and Beyer, 2001; Iwakura et al., 2008; Hasbi et al., 2009; Perreault et al., 2012; Xing et al., 2012).

#### Glial cell-derived neurotrophic factor (GDNF)

GDNF has been shown to promote neurogenesis and has been associated with the beneficial effects of exercise in stroke recovery (Kobayashi et al., 2006; Ohwatashi et al., 2013). As far as we know, whether GDNF expression can be modulated by AMPH administration during poststroke recovery remains to be firmly established. Meanwhile, studies have reported that DA receptor stimulation by direct agonists can augment GDNF levels (Ohta et al., 2004, 2010; Du et al., 2005; Carnicella et al., 2009; Ahmadiantehrani and Ron, 2013), suggesting that DA-enhancing drugs such as AMPH and L-DOPA mediate their beneficial effects on poststroke recovery in part through a modulation of GDNF expression. Indeed, Kuric et al. (2013) showed that L-DOPA treatment caused an increase in GDNF in the infarct core and peri-infarct region in a rat MCAO stroke model. The GDNF increase was attributed to D_1_R-expressing reactive astrocytes which appear in the peri-infarct region around 7 days following stroke. Interestingly, in cell culture, this relationship was only seen following oxygen-glucose deprivation experiments (Ruscher et al., 2012; Kuric et al., 2013).

### Potential recruitment of cocaine- and amphetamine-regulated transcript

Cocaine- and amphetamine-regulated transcript (CART) has been marked as a potential neuroprotective agent, however some data suggests it may be able to mediate poststroke recovery after the acute stage as well (Xu et al., 2006; Jia et al., 2008). CART was found to be lower in the ipsilateral hemisphere of stroke animals following stroke and slowly returned to control levels in a manner that was not modulated by AMPH (Liu et al., 2011). In a MCAO model, mice receiving intranasal administration of CART peptide 3 days poststroke showed improved motor performance, and a number of markers indicating enhanced sprouting of neurons, increased neurogenesis and better vascular recovery (Luo et al., 2013; Liu et al., 2016). CART is unlikely to be the major mediator of the effects of AMPH following stroke (Rogge et al., 2008; Luo et al., 2013). Nonetheless, with its strong interaction with the reward system and the co-localization of CART with DA receptors in some neurons receiving dopaminergic projections, it seems very likely that CART is able to respond to the effects of dopaminergic stimulation (Hubert et al., 2008; Rogge et al., 2008; Hu et al., 2015; Borkar et al., 2018).

### Modulation of immune and inflammatory responses

L-DOPA treatment in the MCAO paradigm was also linked to modulation of the immune and inflammatory response to stroke. The immune response to stroke is complicated, and still poorly understood. It doubtlessly includes elements that are part of endogenous recovery processes, and elements that are maladaptive in overall recovery. Cytotoxic T-cells may be one of these detrimental immune elements, and could contribute to blood-brain barrier permeability, increase inflammation and release of cytotoxins. Animals treated with L-DOPA had lower levels of cytotoxic T-cells (CD3^+^CD8^+^ T-cells), lower levels of pro-inflammatory cytokines IL-5, IFN-⋎, IL-4, and TFN-α in the infarcted hemisphere and lower level of major histocompatibility complex class II (MHC II) cells in the infarct zone (Kuric and Ruscher, 2014a,b). These MHC II cells expressed D_1_R. Meanwhile, L-DOPA treatment was able to ameliorate ischemia-induced lymphocytopenia and increase levels of CD3^+^CD4^+^ T-helper cells in the blood (Kuric and Ruscher, 2014c). Similar to the findings of *de novo* L-DOPA responsiveness in reactive astrocytes, *de novo* expression of D_2_R has been observed on microglia following stroke. These microglia were observed in the infarct area and were responsive to a D_2_R/D_3_R agonist (Huck et al., 2015). In an intracerebral hemorrhage stroke (ICH) model, D_2_R expression in microglia, astrocytes and neurons increased in the perihemorrhage region around 24 h after ICH and stayed high for 7 days poststroke. D_2_R knockdown led to increases in pro-inflammatory cytokines and chemokines in the infarct region. This D_2_R-mediated effect was linked to levels of the anti-inflammatory protein αB-crystallin, which was expressed in a similar pattern as D_2_R following ICH (Zhang et al., 2015). A further study, in a mouse MCAO model, found that the anti-inflammatory bioactive alkaloid sinomenine, was able to reduce inflammatory processes after the stroke, and that this was mediated through an increase in D_2_R expression on astrocytes, as well as an increase in αB-crystallin levels (Qiu et al., 2016).

### Vascular recovery

One region of stroke recovery which is often overlooked is vascular recovery. While endogenous recovery processes do promote angiogenesis, there is evidence that these processes can be modulated. In the wake of stroke it is important to re-establish blood flow, and appropriate blood flow can help support recovery processes (Arai et al., 2009; Liu et al., 2014). As mentioned above, DA has been linked to molecules which show angiogenic effects following stroke, such as FGF-2 and CART. DA receptors are also capable of increasing cerebral blood flow, possibly through a D_1_R mediated pathway, and D_1_R/D_5_R are found on the microvasculature (von Essen, 1974; Krimer et al., 1998; Choi et al., 2006; Tan, 2009; Ohlin et al., 2012). These findings suggest that DA may be able to modulate this aspect of recovery. Indeed, the D_1_-class agonist dihydrexidine has been shown to enhance cerebral perfusion in humans (Mu et al., 2007).

### A pleiotropic role of DA in stroke recovery

**Figure 1:**
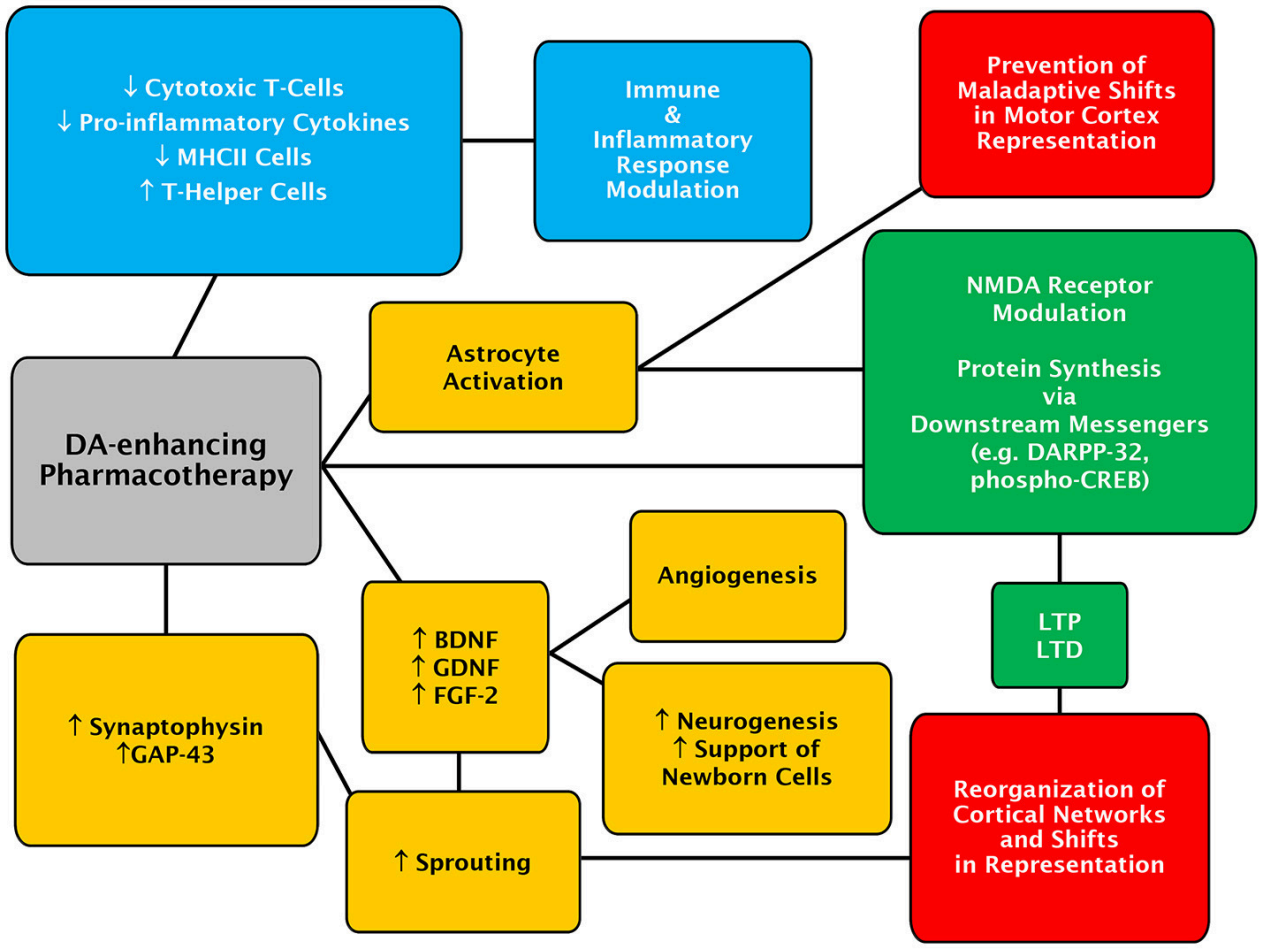
Concept map of the potential mechanisms of action for DA-enhancing pharmacotherapies in poststroke recovery. Mechanisms of actions and effects evoked by DA-enhancing drugs, which are likely to be primarily mediated through D_1_- and D_2_-class receptors, are depicted in colored boxes. The DA-enhancing actions culminating in astrocytic-dependent regulatory processes are in yellow while those involving the recruitment of neuronal signaling partners and modulation of synaptic adaptation are in green. Immune and inflammatory-linked events are in blue. Lastly, changes at the level of cerebral cortex are shown in red.

Given the diverse findings surrounding possible mechanisms of a dopaminergic system-mediated enhancement of recovery following stroke, it seems likely that DA plays a pleiotropic role in stroke recovery processes (Figure 1). While DA may not be the driving force behind reversal of diaschisis, it is implicated in plasticity processes such LTP and long-term depression (LTD), which lead to motor-map reorganization. Recently, an elegant study has demonstrated that activation of the cyclic adenosine monophosphate (cAMP) response element binding (CREB) protein plays an important role in poststroke recovery from motor deficits (Caracciolo et al., 2018). This might also be of functional relevance for a role of DA in recovery from stroke as it can regulate CREB activity (Hyman et al., 1995; Gershon et al., 2007; Belgacem and Borodinsky, 2017). Additionally, DA appears to play a role in sprouting of spared neurons, and support of new born neurons through potentially modulation of growth factor expression. DA is also implicated in the modulation of immune and inflammatory processes and may play a role in promoting angiogenesis. Recovery from stroke is a complicated process, and a pharmacological treatment which is able to modulate multiple aspects of recovery may be beneficial to stroke sufferers.

## Clinical evidence for the use of DA-enhancing drugs in motor recovery from stroke

Results of clinical studies employing DA-boosting drugs to enhance motor recovery following stroke are summarized in Table 2.

**Table 2:** Summary of clinical trials using indirect and direct dopaminergic agonists to improve poststroke motor recovery.

**Study**	**Number of patients**	**Time from stroke**	**Drug dosage**	**Additional rehabilitation**	**Outcome**
Acler et al., 2009	10	10–48 months poststroke	Crossover study design, 100 mg L-DOPA daily or placebo daily for 5 weeks, followed by 2 months of washout and the other condition (single blind)	No physiotherapy	L-DOPA improved walking speeds (10 m walking test), manual dexterity with the paretic hand (9-hole peg test), and no change on the RMA. Increase in cortical silent period as detected by TMS, no changes were seen in the placebo group
Cramer et al., 2009	33	1–12 months poststroke	Daily doses of ropinirole or placebo for 9 weeks, goal to work up to 3 mg/kg with doses adjusted weekly	Physiotherapy given twice a week about 1 h after drug intake from weeks 6 to 9, patients expected to complete 30 min/day of physiotherapy at home after taking medication	No differences were apparent between treatment groups on gait velocity, gait endurance, arm, or leg FM score or BI
Crisostomo et al., 1988	8	≤10 days poststroke	A single 10 mg dose of AMPH	45 min of physiotherapy within 3 h of drug	Statistically significant improvement of the AMPH group above the level of the placebo group by the FM scale
Floel et al., 2005	9	>1 year poststroke	Crossover trial design, with a single dose of 100 mg L-DOPA (with carbidopa) and placebo separated by 24 h	Task specific training 60 min following drug or placebo administration	Improved motor-learning on a TMS stimulated thumb movement task
Gladstone et al., 2006	71 stratified by hemiparesis severity	5–10 days poststroke	10 mg AMPH or placebo	Ten 1-h sessions of physiotherapy given after drug administration	No significant difference in improvement above the level of placebo on the FM scale
Grade et al., 1998	21	Sub-acute stage, specifics unclear	3 weeks of daily MPH, starting at 5 mg and increasing to 30 mg or placebo	Physiotherapy	Significant improvement of the MPH group on the motor portion of the FIM
Kakuda et al., 2011	5	18–143 months poststroke	1 week of prior 100 mg L-DOPA, 15 days of inpatient protocol with continuing L-DOPA and 4 weeks L-DOPA after inpatient protocol, no placebo group	2 daily session of 30 min low frequency TMS applied to the contralateral hemisphere, 1 h of intensive occupational therapy and 1 h of self-exercise during the inpatient protocol	All patients showed improvement in motor function as measured by the FM scale and the Wolf Motor Scale
Lokk et al., 2011	78	15–180 days poststroke	125 mg L-DOPA, 20 mg MPH, 125 mg L-DOPA and 10 mg MPH or placebo, 5 days a week for 3 weeks	Physiotherapy 1 h after drug administration	Significantly better improvement on BI and NIHSS for all drug groups as compared to placebo, but not on the FM scale, at the 6 month follow-up
Martinsson and Wahlgren, 2003	45	≤72 h poststroke	2.5, 5, or 10 mg dose of D-AMPH given orally twice a day, or placebo for 5 days	No additional physiotherapy	Significantly better improvement on LMAC motor function score and AI motor score and SSS-68 at day 7 follow-up in AMPH group, no difference at 1 or 3 months
Masihuzzaman et al., 2011 (abstract only)	97	Unspecified	125 mg L-DOPA or placebo, frequency unspecified	Physiotherapy with drug administration	L-DOPA group had significantly greater increase in RMA as compared to placebo
Mazagri et al., 1995 (abstract only, discussed in Long and Young, 2003)	25	≤72 h poststroke	A single 10 mg dose of D-AMPH or placebo	Physiotherapy	No improvement of AMPH group above the level of placebo group at 48 h or 3 months after treatment in FM scale, BI, and CNS
Platz et al., 2005	26	An average of 5.6 weeks poststroke	10 mg D-AMPH or placebo twice a week for 3 weeks	Arm training 2 h after drug administration	D-AMPH group did not improve above the level of placebo group during or after training, or at 1 year follow-up in TEMPA task (an ADL measure), an aiming task, a finger tapping task and time to walk 10 m
Reding and Borucki, 1995 (abstract only, discussed in Long and Young, 2003)	21	<1 month poststroke	10 mg D-AMPH for 14 days followed by 5 mg D-AMPH for 3 days or placebo	No physiotherapy provided by the study	No benefit of AMPH group as compared to placebo on the FM scale or the BI
Restemeyer et al., 2007	10	>6 months poststroke	Crossover trial design, single dose of 100 mg L-DOPA (with carbidopa and domperidone) and placebo	1 h of physiotherapy after drug administration	No benefit of L-dopa on the 9 hole peg test, grip strength, Action Research Arm Test, or excitability as measured by TMS
Rösser et al., 2008	18	Patients in chronic stage, a mean of 3.3 years poststroke	Crossover study design, comparing 3 doses of 100 mg L-dopa (with carbidopa) or placebo	Drug administration followed by 1 session of procedural motor learning	Better procedural motor learning in the L-DOPA group on a serial reaction time task with a probabilistic sequence in the paretic hand
Scheidtmann et al., 2001	53	Between 3 and 6 months poststroke	100 mg L-DOPA (with carbidopa) or placebo daily for 3 weeks	Physiotherapy daily (with drug) for 3 weeks followed by 3 weeks of only physiotherapy (Monday to Friday)	Significant improvement of the L-dopa group as compared to the placebo group as measured by the RMA
Schuster et al., 2011	16	14–60 days poststroke	10 mg D-AMPH or placebo orally twice a week for 5 weeks	Physiotherapy given after each drug administration	Significantly better improvement on ADL and the arm subscale of the CMSA compared to placebo
Sonde and Lökk, 2007	25	5–10 days poststroke	10 doses 20 mg of d-AMPH over 2 weeks or 10 mg D-AMPH and 50 mg L-DOPA or 100 mg L-DOPA or placebo	Physiotherapy after drug intake	Did not see benefit above the level of placebo on the FM scale or BI for any drug condition
Sonde et al., 2001	39	5–10 days poststroke	10 mg D,L-AMPH or placebo given twice a week for 5 weeks	Physiotherapy given 1 h after each drug administration	AMPH group did not show improvement above the level of placebo on the FM or BI
Treig et al., 2003	24	≤6 weeks poststroke	10 sessions of 10 mg D-AMPH or placebo every 4th day	Physical therapy within 1 h of drug intake	No benefit of AMPH above placebo on the BI or the RMA over course of treatment or at 90 day follow-up
Vachalathiti et al., 2001 (abstract only, discussed in Long and Young, 2003)	27	An average of 5.7 days poststroke	10 mg D-AMPH daily for 7 days	Not stated	No difference between AMPH and control groups on the FM scale and the BI
Walker-Batson et al., 1995	10	Between 16 and 30 days poststroke	10 mg D-AMPH orally every 4th day for 10 sessions (single blind)	Physiotherapy after drug administration	Drug group had significant benefit as compared to placebo at 1 week and 1 year from treatment conclusion as measured by the FM scale
Wang et al., 2014	9	7–30 days poststroke	One time dose of 20 mg liquid MPH, orally or placebo	Transcranial direct current stimulation (tDCS) or sham tDCS	All groups showed improvement on the Perdue Pegboard test, combination MPH and tDCS showed improvement above the level of either treatment alone

*The above table summarizes the studies discussed in the text using amphetamine (AMPH), levodopa (L-DOPA), methylphenidate (MPH) and ropinirole. The following short forms have been used to indicate outcome measures: RMA, Rivermead Motor Assessment; FIM, Functional Independence Measure; BI, Barthel Index; FM, Fugl-Meyer; NIHSS, National Institute of Health Stroke Scale; ADL, Activities of Daily Living; CMSA, Chedoke-McMaster Stroke Assessment; CNS, Canadian Neurological Scale; AI, Activity Index; SSS68, Scandinavian Stroke Scale 68; LMAC, Lindmark Motor Assessment Chart; and the TEMPA task, Test Évaluant les Membres Supérieurs des Personnes Agées, a test of upper extremity function in the elderly*.

### Amphetamine studies

The successful transfer of the beneficial effect of AMPH observed in animal stroke models into medical practice have been hampered by the mixed results obtained in clinical trials. Indeed, an early study with eight stroke patients showed that a single dose of AMPH followed by physical therapy could significantly increase motor function improvement above the level attained with placebo and physical therapy (Crisostomo et al., 1988). Meanwhile, another preliminary study in 25 patients found a single dose of AMPH was not sufficient to improve motor function recovery above the level of placebo treatment (Long and Young, 2003). Some studies employing repeated dosing and physiotherapy, usually at an interval of several days, have shown a significant benefit of AMPH on motor recovery and/or activities of daily living, particularly in the hemiparetic arm (Walker-Batson et al., 1995; Martinsson and Wahlgren, 2003; Gladstone et al., 2006; Schuster et al., 2011). Other trials using similar dosing patterns have found no benefit (Sonde et al., 2001; Treig et al., 2003; Platz et al., 2005; Gladstone et al., 2006; Sonde and Lökk, 2007). More aggressive dosing approaches, with daily AMPH, have yet to produce positive results (Long and Young, 2003). Unfortunately, studies supporting the use of AMPH for motor recovery often have low enrollment. Experimental group dissimilarity is also a concern, with many studies showing differences in mean patient age or in level of motor function after stroke, across both successful and unsuccessful trials. A 2007 meta-analysis of six studies (176 patients) that looked at motor recovery saw evidence of better relative change from poststroke baseline in patients given AMPH and a nearly significant effect of AMPH on activities of daily living score (Martinsson et al., 2007). A later meta-analysis did not observe a significant improvement of motor scores in patients given AMPH, although the trend for an increased motor benefit was still present (Sprigg and Bath, 2009).

### L-DOPA studies

Studies employing L-DOPA for motor recovery from stroke have had somewhat more success, although results are still mixed. In the sub-acute phase several studies showed L-DOPA and physical therapy improve recovery above the level of placebo and physical therapy (Scheidtmann et al., 2001; Lokk et al., 2011; Masihuzzaman et al., 2011). One study was only able to find a trend toward improvement with L-DOPA, although this group had only 4 patients in the L-DOPA group, and 7 patients in a combination L-DOPA and AMPH group, as well as a high degree of initial variability and an uneven distribution of deficits on the Fugl-Meyer arm portion (Sonde and Lökk, 2007). The study by Masihuzzaman et al. (2011) reported that both ischemic and hemorrhagic strokes responded well to the L-DOPA treatment. In the chronic phase a single dose of L-DOPA was sufficient to significantly boost motor learning on a TMS-induced thumb movement paradigm (Floel et al., 2005), but a single dose could not boost performance on the Nine-hole Peg Test, the Action Research Arm Test or on grip strength (Restemeyer et al., 2007). The study by Floel et al. (2005) trained participants on the test task, whereas, the Restemeyer et al. (2007) study employed a physiotherapy session with an emphasis on dexterity. These studies possibly suggest that a single dose is not enough to lead to generalizable motor improvement in a chronic stroke population, although it should also be noted that there was substantial variability in scores for the Nine-hole Peg test, and that performance on the Action Research Arm test (a test which is prone to ceiling effects) was high before intervention (Restemeyer et al., 2007; Duncan, 2013). In support of multiple doses being required for enhancing stroke recovery, a group of patients received three doses of L-DOPA over 2 days which led to increased procedural motor learning in the paretic hand without changes to motor arousal or response style (Rösser et al., 2008). Moreover, 5 weeks of L-DOPA without physiotherapy ameliorated scores on the Nine-hole Peg test and augmented speed of 10-meter walking, as well as lengthening the cortical silent period, while no change was seen in placebo group. Although the study was not able to show improvement on the Rivermead Motor Assessment, these findings indicate that L-DOPA alone could be sufficient to improve motor recovery and modulate cortical excitability (Acler et al., 2009). While the above findings do suggest that there is a beneficial role for L-DOPA in poststroke motor recovery, the trials performed thus far are not without flaws. Several of the trials have involved few patients or have used a crossover design, and one study only employed single blinding. Two large scale clinical trials called “Dopamine Augmented Rehabilitation in Stroke” (DARS) (Bhakta et al., 2014) and “Effect of Serotonin and Levodopa in Ischemic Stroke” (SELEIS, ClinicalTrials.gov identifier: NCT02386475), using L-DOPA and physical therapy in stroke patients are underway and perhaps will be able to give further insight into the efficacy of this treatment approach.

There has also been interest in combining L-DOPA pharmacotherapy with other emerging recovery strategies. One group tested an aggressive treatment plan involving a total of 7 weeks of daily L-DOPA, and 15 days of inpatient care that involved 20 min of low frequency TMS applied to the contralateral hemisphere, 1 h of one-on-one occupational therapy and 1 h of self-exercise, repeated twice daily, on chronic stroke sufferers. All patients in the small cohort showed improved motor function in the paretic arm, most of which was maintained at the 4 week follow up, however the absence of any control groups makes drawing any conclusions difficult (Kakuda et al., 2011). Lastly, the use of L-DOPA to facilitate robot-assisted motor therapy following stroke has also been proposed (Tran et al., 2016).

### Methylphenidate (MPH) studies

The use of MPH to enhance stroke recovery has also been tested in clinical studies. One to three weeks of MPH combined with physical therapy significantly increased scores on the modified functional independence measure (FIM) and nearly significantly increased performance on the Fugl-Meyer scale in post-acute stroke patients (Grade et al., 1998). Moreover, Lokk et al. (2011) found that MPH and MPH + L-DOPA were able to improve scores on the Barthel Index and National Institute of Health Stroke Scale, but not on the Fugl-Meyer Scale, as compared to placebo treatment. Further, another study reported a single dose of 20 mg of MPH was able to improve performance on a finger tapping task, though not performance of grip strength or a target pursuit task (Tardy et al., 2006). The improvement in finger tapping was correlated to hyperactivity of the ipsilateral primary sensorimotor cortex and the contralateral premotor region, as measured by functional MRI during the drug's active period. A hypoactivity of the anterior cingulum was also observed under the MPH condition (Tardy et al., 2006). Interestingly, the combination of MPH and TMS led to significant motor benefits, which were above the level of either treatment alone on the Perdue Peg Board test (Wang et al., 2014). However, as no placebo group was included in this study, it is unclear if the modest improvement seen in peg board performance in the MPH alone group is of any potential clinical relevance. Likewise, while this study did not show changes in cortical excitability in any of their MPH or TMS groups, it is worth mentioning that MPH has been shown to regulate cortical excitability during a motor task in healthy patients (Kratz et al., 2009; Wang et al., 2014).

### Direct dopamine agonists

To date only one study tested a direct dopamine agonist, the D_2_-class drug ropinirole, along with physical therapy to facilitate motor recovery from stroke (Cramer et al., 2009). In spite of all groups showing significant improvement over the study period, there was no benefit of ropinirole treatment in either recent (earlier than 3 months from onset) or later (between 3 and 12 months from onset) stroke cases. Importantly, ropinirole showed no serious adverse effects, and medication compliance was high, yet the majority of patients in the drug group never reached the planned dose (3 mg daily) for the study. Additionally, the drug group had a higher rate of comorbidities such as diabetes and depression, and the placebo group received a higher level of physiotherapy outside of the parameters of the study (Cramer et al., 2009).

Further studies in human stroke patients, examined drugs given to stroke patients for co-morbidities, classifying them as “potentially deleterious” or not, based on preclinical findings. The “potentially deleterious” group, which included dopamine antagonists, were found to worsen motor outcome (Goldstein and Davis, 1990a; Goldstein, 1995, 1998).

### Why have clinical results been so mixed?

There are many challenges inherent in translating results from animal studies into human patients, particularly in the stroke field (Corbett et al., 2017). Animal studies are typically done in young, otherwise healthy animals without comorbidities or other medications. Animal models have the benefit of producing extremely similar strokes in animals with identical genetic backgrounds. In clinical stroke studies factors such as age or stroke severity can have a greater impact on outcome than the expected benefit of a given treatment, so groups must be well balanced, or analysis should take co-variants into account (Bath et al., 2012). Furthermore, appropriately scaling factors such as dosage, timing and rehab intensity from animal to human models is likely to be of critical importance, but may not be straightforward (Adkins et al., 2009). Consider the issue of timing after stroke. While humans typically show spontaneous recovery over the 6 months following stroke, rodents see spontaneous recovery plateauing at about 4 weeks following stroke, however it is difficult to determine what the timing of the physiological recovery processes observed in animals would look like in humans (Chollet et al., 2014).

#### Stroke lesion location

Aside from the intricacies of dose, timing and rehab type and intensity, one potential problem as to why studies have had such mixed results is that studies may not be targeting the responsive patient populations (Chollet et al., 2014), nor are the studies large enough to allow for stratification of patient populations. In terms of characteristics of the stroke, responsiveness to pharmacotherapy could depend on time from onset of stroke, severity of deficit or stroke location. Stroke location should be considered carefully. One animal study showed that AMPH worsened outcome in rats given unilateral nigrostriatal pathway lesions (Mintz and Tomer, 1986). This may have relevance in patients who present with delayed substantia nigra degeneration ipsilateral to the infarct. For these patients an approach using direct agonists may be better than indirect agonists which could potentially culminate in higher risk of DA depletion. One study has reported that patients whose stroke occurs on their dominant side respond better to dopaminergic pharmacotherapy. This is believed to occur because the dominant hemisphere, equally boosted by the pharmacotherapy, may overpower and inhibit the infarcted, non-dominant hemisphere. As discussed in section Cortical Map Reorganization, increased assumption of function by the contralateral hemisphere is considered undesirable recovery strategy (Rösser and Flöel, 2008; Boyd et al., 2017). That is not to say that such patients cannot benefit from pharmacotherapy, however, they may also need to engage other strategies, such a cortical inhibition of the unaffected hemisphere using TMS, or CIMT to ensure optimal recovery.

#### Genetic polymorphisms

Another factor that may be a source of a lot of variation between studies is the genetics of the individuals involved. Research suggests that polymorphisms in genes such as BDNF and apolipoprotein E may influence stroke recovery, and specifically may interact with treatments (Stewart and Cramer, 2017). Genetic polymorphisms potentially impacting dopaminergic response exist at varying rates in the population notably for COMT, DAT, D_1_R, D_2_R and D_3_R (Pearson-Fuhrhop et al., 2013). Taking into account the well-established inverted U-shaped dose-effect relationship to DA levels on a variety of behavioral outputs, the response of a patient to DA modulating treatments may differ greatly based on where their genetics place them on the curve (Cools and D'Esposito, 2011; Thirugnanasambandam et al., 2011; Floresco, 2013; Vaillancourt et al., 2013; Arnsten et al., 2015). Evidence linking variations in the gene for COMT to differences in motor skill outcome in stroke patients, given regular physical therapy treatment, suggests that baseline “dopaminergic tone” is one factor in recovery (Liepert et al., 2013; Kim et al., 2016). Thus, the genetics of the individual may also interact with DA-enhancing treatments. DAT polymorphisms have been shown to impact the response of children with attention deficit hyperactivity disorder (ADHD) to MPH in both behavioral and neurophysiological measures (Stein et al., 2005; Gilbert et al., 2006). Several studies in healthy adults have shown that use of drugs increasing DA levels are beneficial on motor learning, impulse control, and working memory tasks in participants whose genetics make them prone to a lower dopaminergic tone (Mattay et al., 2003; Pearson-Fuhrhop et al., 2013; MacDonald et al., 2016). In contrast, participants with a higher dopaminergic tone perform better at baseline, but see their performance worsen under the influence of DA-enhancing drugs (Mattay et al., 2003; Pearson-Fuhrhop et al., 2013; MacDonald et al., 2016). While most studies examine their results by single genetic factors, an emerging and promising approach is the use of a gene score, which assigns a direction of impact on the level of dopaminergic system activity to each polymorphism and evaluates participants by where they fall on a combined score (Pearson-Fuhrhop et al., 2013). This approach allows one to account for the combined effects of various polymorphisms. However, the direction of the predicted effect of the polymorphism on dopaminergic tone should be carefully investigated for all the genes involved, and the role of the gene in the specific process under investigation must be considered. Recently the COMT gene rs4680 single nucleotide polymorphism that changes the wild type G allele (Val) to an A allele (Met) at amino acid 158 (Vat158Met) has been assessed with respect to motor learning under L-DOPA using this gene scoring approach (Pearson-Fuhrhop et al., 2013). Pearson-Fuhrhop et al. (2013) assigned individuals who are homozygous for the G allele (Val) a score of 0 for that gene, and individuals with the A allele a score of 1, because the Met variant of COMT has a lower activity and therefore people with this gene will have greater extra-synaptic levels of DA. Interestingly, they found that their gene-score model fit better to the motor learning under L-DOPA results obtained, when the score for the COMT gene was taken out of account (Pearson-Fuhrhop et al., 2013). The logic behind this interpretation of the COMT polymorphism seems sound when you consider that the A allele of the COMT gene (Met variant) was associated with improved baseline performance, and worse drug-influenced performance on pre-frontal cortex based tasks, however, in stroke patients not given drugs, better motor performance was seen in patients with the COMT Val/Val or Val/Met genotypes (Mattay et al., 2003; Liepert et al., 2013; Kim et al., 2016). Levels of COMT are higher in the prefrontal cortex, and lower in the striatum, while the opposite is true of DAT (Mattay et al., 2003). As a result, higher prefrontal DA levels may lead to increased prefrontal cortex inhibition of striatal activity, without concomitant increases in striatal DA, so in motor learning based tasks, which require striatal and motor cortical activity, the Met allele of the COMT gene may not be beneficial. Adding to the complexity of considering whole-network ramifications of the genes governing the dopaminergic system, there is evidence that polymorphisms in the BDNF gene may interact with polymorphisms for DA-linked genes and affect plasticity processes (Witte et al., 2012). A great deal of work remains to be done on the pharmacogenetics of stroke recovery, particularly as it pertains to the dopaminergic system.

#### Spectrum of actions of DA-enhancing drugs

Studies thus far have mostly used drugs that act upon a wide variety of targets. By virtue of a modulation of the DAT and NE transporter function, AMPH and MPH can potentially activate numerous adrenergic (α_1_AR, α_2_AR, β_1_AR, β_2_AR) and dopaminergic (D_1_R, D_2_R, D_3_R, D_4_R, and D_5_R) receptor subtypes. Another confounding factor with respect to the usage of these drugs is the potential recruitment of the trace amine-associated receptor 1 (TAAR1), a Gs-linked GPCR widely expressed in the central nervous system (CNS). Notably, TAAR1, which chiefly responds to endogenous trace amines (β-phenylethylamine, tyramine, and octopamine), can also bind to and be activated by DA, NE and AMPH-like compounds (Pei et al., 2016). Likewise, administration of L-DOPA, the precursor to DA and NE, possibly leads to a non-specific activation of a large number of CNS dopaminergic and adrenergic receptors. Consequently, the potential recruitment of a large spectrum of molecular targets by these drugs may ultimately mitigate their therapeutic effects. For instance, D_1_ and D_2_-class dopaminergic receptors act in opposing directions in their classical pathways. Previous pharmacological manipulations of the dopaminergic system, for example the use of D_2_-class receptor antagonists in the treatment of psychosis, have shown that drugs preferentially targeting a given receptor or receptor-class are often more efficacious to treat symptoms without causing unwanted side effects (Beaulieu et al., 2015). It is likely that the best dopaminergic facilitation of stroke recovery comes from some optimal balance between D_1_ and D_2_-class receptor activation, and it is possible that a non-specific increase in DA receptor stimulation may not allow reaching that optimal balance. Several of the likely mechanisms of action for a DA-driven facilitation of stroke recovery could be particularly associated with D_1_-class dopaminergic receptors, including the modulation of motor learning, augmentation of BDNF and GDNF levels and increase in cerebral blood flow and neurovascular coupling (Luft et al., 2004; Choi et al., 2006; Nitsche et al., 2009; Tan, 2009; Perreault et al., 2012; Xing et al., 2012; Kuric et al., 2013). Specific activation of D_1_-class receptors would be unlikely to have great effects on endogenous DA levels, as feedback inhibition is the domain of D_2_-class receptors, particularly presynaptic D_2_R (Boyar and Altar, 1987; Beaulieu et al., 2015). Conversely, D_2_-class receptors seem to be linked to modulation of immune responses (Huck et al., 2015; Zhang et al., 2015; Qiu et al., 2016), LTP/LTD in concert with D_1_-class receptors (Calabresi et al., 2007; Shen et al., 2008; Molina-Luna et al., 2009) and it has been suggested that these receptors can also modulate relevant growth factors, BDNF (Takeuchi et al., 2002; Ahmadiantehrani and Ron, 2013; Rioult-Pedotti et al., 2015; Adachi et al., 2018), GNDF (Bozzi and Borrelli, 1999; Ohta et al., 2010) and FGF-2 (Roceri et al., 2001; Fumagalli et al., 2003, 2006b; Li et al., 2006; Mueller et al., 2006). Notwithstanding these findings, the intricate role of biogenic amines in the facilitation of poststroke recovery, notably that of DA and its receptors, remains to be experimentally tested in a more systematic fashion. This is likely to improve our understanding of the mechanistic underpinnings that underlie stroke recovery, which in turn may lead to better treatment in the future.

### Potential complications to DA-enhancing therapies in stroke patients

As mentioned previously, following stroke there is a massive release of DA, which is thought to be a source of excitotoxicity, and to exacerbate cell damage. As such, it is likely that dopaminergic therapies should be avoided in the acute stages of stroke and are best applied once the stroke has stabilized (Goldstein, 1998). As alluded above, to improve stroke recovery these therapies may be best in the context of personalized medicine, as patients with the wrong genotype or stroke locations may not benefit from these therapies. Another group of patients who may be harmed from a dopaminergic therapy are the rare individuals who develop poststroke movement disorders and may not respond well to DA-enhancing therapy (Handley et al., 2009; Siniscalchi et al., 2012; Ruppert et al., 2017).

Additionally, administration of AMPH and the related methamphetamine can lead to degeneration of DA cells and terminals in a variety of rodent brain regions including the striatum and the olfactory bulb (Deng et al., 2007; Atianjoh et al., 2008). In fact, use of high doses of AMPH and derivatives has been shown to lead to neurotoxicity in rodent models, culminating in a decrease in striatal DA, which can last for years after the cessation of the drug (Berman et al., 2008, 2009; Yamamoto et al., 2010). Interestingly, nonhuman primates administered with lower and more clinically relevant AMPH doses display the neurotoxicity hallmarks seen in rodents given high doses, suggesting humans may be more susceptible to this phenomenon than rodents (Ricaurte et al., 2005; Berman et al., 2009). Further, it has been suggested that elderly individuals may be more vulnerable to this effect, which perhaps emphasizes the non-ideal nature of AMPH treatment, should other agents prove suitable for stroke recovery (Berman et al., 2009). However, the clinical relevance of this potential neurodegeneration induced by AMPH derivatives in the context of poststroke recovery has yet to be confirmed as per the dose regimens (AMPH) and type of drugs used (therapeutic usefulness of methamphetamine, if any, is probably limited). In the following sub-sections we discuss the more potentially clinically relevant unwanted effects of drugs utilized in the context of improving poststroke recovery. The reader should bear in mind that most dopaminergic drugs referred herein have been also used to treat some neuropsychiatric disorders (e.g., PD, ADHD). Importantly, whether some of undesirable effects (e.g., impulse-control disorders) reported for instance in PD (Weintraub et al., 2010; Mestre et al., 2013; Maloney et al., 2017; Voon et al., 2017) could also be observed with “sub-chronic” time course employed in clinical stroke recovery studies (see Table 2 for drug scheduling used with approved dosage by US Food Drug Administration), is unclear.

#### AMPH and MPH

The use of AMPH has its own particular risks, most notably cardiovascular side effects, nervousness, insomnia, and loss of appetite, and is not considered an ideal therapy for these reasons (Martinsson and Wahlgren, 2003; Floel and Cohen, 2010; Engelter, 2013). Likewise, although MPH is typically well tolerated, common side effects include hypertension, insomnia and anorexia. Meta-analysis of the clinical stroke data indicates a trend toward increased risk of death in AMPH-treated patients (Martinsson et al., 2007; Sprigg and Bath, 2009). However, no single study reported an increased risk of death, nor any significant adverse effects attributed to AMPH (Martinsson et al., 2007). Several studies did show increases in heart rate and hypertension under the effects of the drug. Considering that these trials used careful screening to avoid treating patients with any additional cardiovascular risk factors, this may mean that AMPH treatment is likely to be unsafe in the broader population of stroke patients (Martinsson et al., 2007; Sprigg and Bath, 2009; Engelter et al., 2010). While studies using MPH after stroke have not shown increased risk of death or side effects under the drug, few trials have been done. MPH studies have had very low ratios of enrollment: screening, indicating strict screening measures are being employed, and again suggesting a limited applicability notably due to cardiovascular risks in older poststroke patients (Engelter, 2013). In support of higher incidence of cardiovascular risks in stroke patients, a recent histological study reported heart and kidney damages in adolescent rats receiving 2 daily administration of the equivalent clinical dose of MPH for 7 days (Loureiro-Vieira et al., 2018).

#### L-DOPA

Side effects of L-DOPA are generally milder. L-DOPA is typically given with dopa-decarboxylase inhibitors to maximize the amount of L-DOPA available in the brain, which may also help to prevent the development of side effects in the peripheral system (Barbeau et al., 1972; Politi et al., 2018). The most common side effect of L-DOPA treatment is nausea, which can be treated with domperidone (Restemeyer et al., 2007; Engelter, 2013). While L-DOPA treatment for Parkinson's disease can lead to dyskinesia, this complication appears to be an interaction with the parkinsonian state, and there has been no reports of dyskinesia developing in stroke patients, even with up to 7 weeks administration, suggesting that dyskinesia is not likely to occur in stroke sufferers unless they also have a Parkinson's disease-like condition (Kakuda et al., 2011). An observational study of the use of drugs for pharmacological augmentation (PA) of stroke recovery found that in one Swiss stroke rehab ward, 39% of patients were being given drugs for PA, of those 65% received L-DOPA. These patients taking PA agents had only mild and transient side effects and the use of PA agents was associated with a larger change in FIM score (Engelter et al., 2010). The fact that L-DOPA is being given quite frequently off label to stroke patients should further spur on studies in this area to confirm efficacy and safety. Overall L-DOPA appears to be relatively safe, with side effects being mild and transient.

#### Ropinirole

Tolerability of ropinirole is less clear. Although it was seemingly safe in the Cramer et al. (2009) study reported above, it did lead to higher levels of side-effects (dizziness, sleepiness, fatigue), and patients were not able to reach the planned dose for the study. Additionally, medication effects overcame the blinding (Cramer et al., 2009).

#### Drug abuse and impulse-control disorders (ICDs)

In studies using ropinirole, L-DOPA or amphetamine derivatives, there has not been any specific observation of DA dysregulation syndrome (a form of ICD) in patients taking dopaminergic drugs during stroke recovery. Meanwhile, some studies have reported that some impulse control disorders (e.g., compulsive shopping or gambling) are possible in non-PD patients (e.g., restless leg syndrome, fibromyalgia, prolactinomas) taking DA agonists (Mestre et al., 2013; Maloney et al., 2017). However, as far as we know, there are no reports of ICDs in non-PD patients taking L-DOPA. With regards to the use of AMPH derivatives, there are no comprehensive studies published specifically about the prevalence of drug abuse or ICDs among patients prescribed with AMPH derivatives to treat neuropsychiatric disorders such as ADHD, for instance. Yet, addiction may be a particular concern, as some evidence suggests that following stroke, there may be an increased vulnerability to addiction, although it is unclear if this is caused by the stroke itself or by an interaction of stroke and prior experience with addictive substances (Huang et al., 2017). Abuse of stimulants is fairly common with non-ADHD adolescents and while rare, there is a risk with ADHD adolescents (Jaffe, 2002). MPH has also been abused, and may be addictive, particularly when administration strays from the prescribed routes and dosages (Challman and Lipsky, 2000; Morton and Stockton, 2000). In adults treated for ADHD (>25-year old), co-medication with other CNS drugs is frequent, and hence will likely confound, if any, the prevalence of drug abuse (Pauly et al., 2018). The incidence of unwanted effects (e.g., drug abuse) with the use of DA-enhancing drugs will also likely depend on demographic, premorbid personality type (e.g., impaired executive function) and/or genetic/epigenetic risk factors (Mestre et al., 2013; Maloney et al., 2017; Voon et al., 2017). Importantly, as discussed in section Genetic Polymorphisms, genetic risk factors associated with DA-related genes have been implicated in the extent of dopaminergic stimulation.

## Conclusions

The interplay of stroke and the dopaminergic system is deeply complex. Strokes can exert significant effects on the DA system, which may require appropriate treatment. An understanding of these effects can set the stage for a better understanding of the effects of dopaminergic treatment following stroke. Results in humans have been mixed but, given to the responsive patient populations, this may be a powerful adjunct to physical therapy. Although beyond the scope of this review, there is also evidence that DA-based pharmacotherapy may be beneficial in aphasia (Gill and Leff, 2012), hemi-spatial neglect (van der Kemp et al., 2017), and poststroke depression (Kohno et al., 2010; Delbari et al., 2011; Adam et al., 2013; Spiegel and Chatterjee, 2014; Stanfill et al., 2016). Meanwhile, like the findings for DA-based pharmacotherapies in motor recovery, results in these areas are quite mixed. The current body of literature provides hope for the efficacy of DA-enhancing drugs for motor recovery from stroke. Studies performed using animal stroke models and in clinical stroke patients seem to suggest an approach pairing multiple administrations of DA-enhancing therapies and intensive physical rehabilitation is the most efficacious. This should be seen as the jumping off point for further studies, at both the preclinical and clinical levels, to optimize issues such as timing and the most responsive patient populations, which will maximize the chances of successful translation. We have identified several possible mediators of this DA system driven effect, including modulation of long-term potentiation and depression, motor map reorganization, growth factors such as BDNF, GDNF, and FGF-2, increased cortical sprouting and interactions with astrocytes and glial cells. Understanding of the underlying mechanisms will allow the field to better choose and design pharmaceutical treatments. Indeed, specific targeting of receptor subtypes may allow for better outcomes, by allowing for manipulation of the most powerful recovery mechanisms, while avoiding undesirable side-effects. More work remains to be done at both the basic science level, to illuminate mechanisms and clarify best practices, and at the clinical level, to solidify the best interpretation of the results in human patients.

## Author contributions

AG and MT contributed to the conception, writing and editing of the manuscript. AG and MT approved the manuscript for publication. AG designed the figure and tables.

### Conflict of interest statement

The authors declare that the research was conducted in the absence of any commercial or financial relationships that could be construed as a potential conflict of interest.
